# A wavelet-based approach generates quantitative, scale-free and hierarchical descriptions of 3D genome structures and new biological insights

**DOI:** 10.1371/journal.pcbi.1013887

**Published:** 2026-01-20

**Authors:** Ryan Pellow, Josep M. Comeron

**Affiliations:** 1 Department of Biology, University of Iowa, Iowa City, Iowa, United States of America; 2 School of Biological Sciences, University of Utah, Salt Lake City, Utah, United States of America; 3 Interdisciplinary Program in Genetics, University of Iowa, Iowa City, Iowa, United States of America; Chinese Academy of Science, CHINA

## Abstract

Eukaryotic genomes are organized within nuclei in three-dimensional space, forming structures such as loops, topologically associating domains (TADs), and chromosome territories. This 3D architecture impacts gene regulation and development, stress responses, and disease. However, current methods to infer these 3D structures from genomic data have multiple drawbacks, including varying outcomes depending on the resolution of the analysis and sequencing depth, qualitative outputs that limit statistical comparisons, and insufficient insight into structure frequency within samples. These challenges hinder rigorous comparisons of 3D properties across genomes, conditions, or species. To overcome these issues, we developed WaveTAD, a wavelet transform-based method that provides a resolution-free, probabilistic, and hierarchical description of 3D organization. WaveTAD generates TAD strengths, capturing the variable frequency of intrachromosomal interactions within samples, and shows increased accuracy and sensitivity over existing methods. We applied WaveTAD to multiple datasets from *Drosophila*, mouse, and humans to illustrate new biological insights that our more sensitive and quantitative approach provides, such as the widespread presence of embryonic 3D organization before zygotic genome activation, the effect of multiple CTCF units on the stability of loops and TADs, and the association between gene expression and TAD structures in COVID-19 patients or sex-specific transcription in *Drosophila*.

## Author summary

Human cells package meters of DNA into micron-sized nuclei through a series of coordinated multiscale looping structures that compose the 3D nuclear organization. At the same time, this complex compression requires dynamic and precise control to maintain biological processes in a manner that safeguards developmental and tissue-specific programs. Aberrations in this undertaking underlie diseases, aging, and cancer. Despite the vital importance of the 3D nuclear organization and regulation, methodologies to infer it robustly and quantitatively from sequencing data are still lacking. To address this need, we developed WaveTAD, a wavelet transform-based method to identify DNA looping structures and assign quantitative strengths in a resolution-free manner. We demonstrate that these strengths correlate with the frequency with which such structures occur in samples and that our methodology is robust to noise and reproducible across biological and technical replicates. The application of WaveTAD to publicly available fruit fly, mouse, and human datasets revealed key insights into the establishment and maintenance of DNA looping structures, as well as the interplay between these structures and gene expression.

## Introduction

The primary linear structure of genetic information within nuclei undergoes a highly specific and programmed multi-level hierarchical organization in three-dimensional (3D) space. These 3D genomic structures, including loops, topologically associating domains (TADs) and territories, facilitate interactions between distant sequences, such as enhancers and promoters, which are vital for orchestrating transcriptional programs in a developmental and tissue-specific manner [1,2]. They also position specific genomic sections in differentially accessible nuclei compartments. Alterations in these tissue-specific 3D structures have been linked to developmental abnormalities, diseases, and cancer [3–8]. However, not all gene expression control is dependent on TAD and loop formation, as some genes show robust expression independent of TAD perturbations [9,10].

Developments in genomic methods, including several forms of high-throughput chromatin conformation capture (Hi-C) [11] and more recently single-cell Hi-C methods [12], allow generating high-resolution DNA sequence data that identify contacts between distant genomic regions. These contacts form matrices (contact matrices), which can be used to infer proximity in 3D space and predict structures such as DNA loops and TADs [13]. Both loops and TADs are units of high-contact density areas within these contact matrices, with TADs showing higher frequency of internal interacting areas relative to loops [14]. However, the raw information used for contact maps is considerably sparse and noisy due to the limited number of informative reads per kilobase (kb) and the presence of random contacts. In the last decade, many computational and mathematical tools have been developed to infer loops and TADs based on contact matrices [15–18]. All these tools integrate contact data at a given genomic scale or resolution that is decided *a priori*, often determined by the size of the genome and number of informative reads. Although high-resolution Hi-C studies do exist [19,20], most studies based on human or mouse bulk Hi-C use a 50kb resolution, analyzing all the reads that connect sites within a specific 50kb genomic region with any other 50kb region across the genome. Studies in species with smaller genomes, such as *Drosophila*, tend to use finer resolutions (e.g., 10–25kb) due to the ease of obtaining higher numbers of informative reads per genomic unit. The collapse of data when using broad resolutions limits the precise identification of contact locations and overlooks weaker secondary contacts within a region, while studies at finer scales generate noisier signals. These limitations are drastically intensified in single-cell studies, often requiring resolutions at or above 500kb [21,22]. Notably, all current methods to infer 3D structures based on contact matrices predict different sets of loops and TADs (number, size and locations) depending on the resolution used in the analyses, restricting biological insights from comparisons between studies and/or species. Another limitation of 3D algorithms is the difficulty in providing quantitative descriptions, further limiting accurate comparisons between structures across genomes or samples.

To address the drawbacks of current methodological approaches to infer the 3D genome organization, we developed a method and a corresponding bioinformatics pipeline to analyze contact frequencies using wavelet-transforms (WT). Proposed in the early 1900s, the popularity of WT began to take hold in the 1980s in physics and geology as a means of signal and, later, image processing [23–26]. When applied to genomic studies, WT can be used to study how any type of signal changes along chromosomes by decomposing it into a set of scaling and wavelet functions [27–29]. These functions provide local information for both location and scale (size of the genomic region) without the need to specify the scale of interest *a priori*, making them scale or resolution-free. Specifically, WT produces detail coefficients that represent the change in signal intensity between neighboring locations at a given scale while removing the noise and signal identified at smaller scales without loss of information. This makes it useful for extracting multi-scale signals from noisy or sparse data [23,30–33] and, for most genomic studies, WT would identify signals starting from the smallest possible scale—a single nucleotide. Importantly, detail coefficients are normally distributed, allowing for the assignment of independent probabilities for each denoised scale-by-location datapoint [23] and the quantitative comparison between signals regardless of scale.

While WT has been used in genomic applications in the past, mostly with the objective of denoising data before downstream analysis [34,35], we propose that WT is an ideal and statistically rigorous framework to analyze contact frequencies across genomes and infer multilevel structures including, but not limited to, loops, TADs, subTADs, and corner-dot TADs. Here, we describe the new method and the accompanying software package (WaveTAD) to analyze Hi-C contact data at the nucleotide level in a probabilistic, resolution-free, and hierarchical manner. We also apply WaveTAD to multiple datasets, both bulk and single-cell Hi-C, to demonstrate its benefits providing new biological insights.

## Results

### Overview of WaveTAD

**Fig 1:**
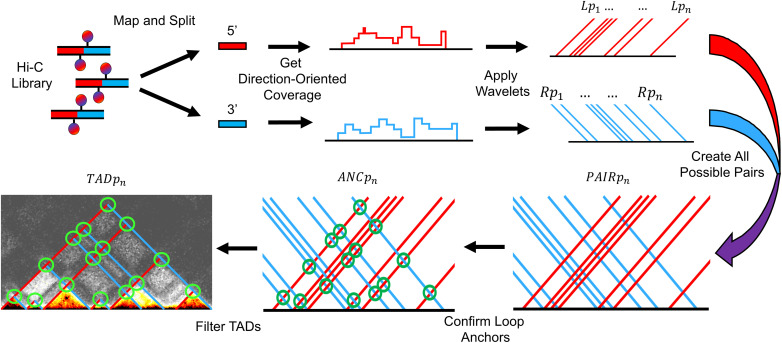
Overview of WaveTAD method.

WaveTAD leverages the statistical and multiscale capabilities of WT to decompose the sparse and noisy Hi-C data to extract valuable information regarding the state of the 3D nuclear architecture (**Fig 1**; see **Methods** for details). Unlike traditional TAD callers that analyze binned contact matrices at specific broad resolutions, WaveTAD probes the raw and underlying coverage signal of valid Hi-C contacts (paired-end sequencing reads uniquely mapping to two distal areas in the reference genome) (**Fig 1A**). Based on the orientation of the mapped reads, they are split into 5’ and 3’ groups indicating contacts downstream and upstream, respectively (**Fig 1B**), and genome-wide nucleotide coverage is calculated independently for the 5’ and 3’ groups (**Fig 1C**). These 5’ and 3’ read depth coverages are used as signal along chromosomes for WT to identify genomic locations displaying sharp increases in distal contacts that represent potential left and right TAD and loop boundaries. True boundaries are then determined by calculating detail coefficients and corresponding probability values across scales after adjusting for multiple comparisons (L*p*_n_ and R*p*_n_ for 5’ and 3’ groups, respectively; **Fig 1D**). To identify TADs and loops, left-side and right-side TAD boundaries are paired to create all possible TADs/loops, with a probability (PAIR*p*_n_; **Fig 1E**) equal to the product of L*p*_n_ and R*p*_n_. These candidate TADs/loops are then filtered based on the presence of long-distance interactions at the predicted TAD apex or loop anchor locations (depicted by the green circles). This is investigated by probing enrichment of contacts relative to a background surrounding the predicted apex/anchor region and by assigning an associated probability to that enrichment (ANC*p*_n_; **Fig 1F**). Finally, the product of the probabilities of the left TAD boundary (L*p*_n_), right TAD boundary (R*p*_n_), and TAD apex or loop anchor (ANC*p*_n_) are calculated and denote the strength (TAD*p*_n_; **Fig 1G**), as a measure of frequency or stability, of the called TADs/loops.

**Fig 2:**
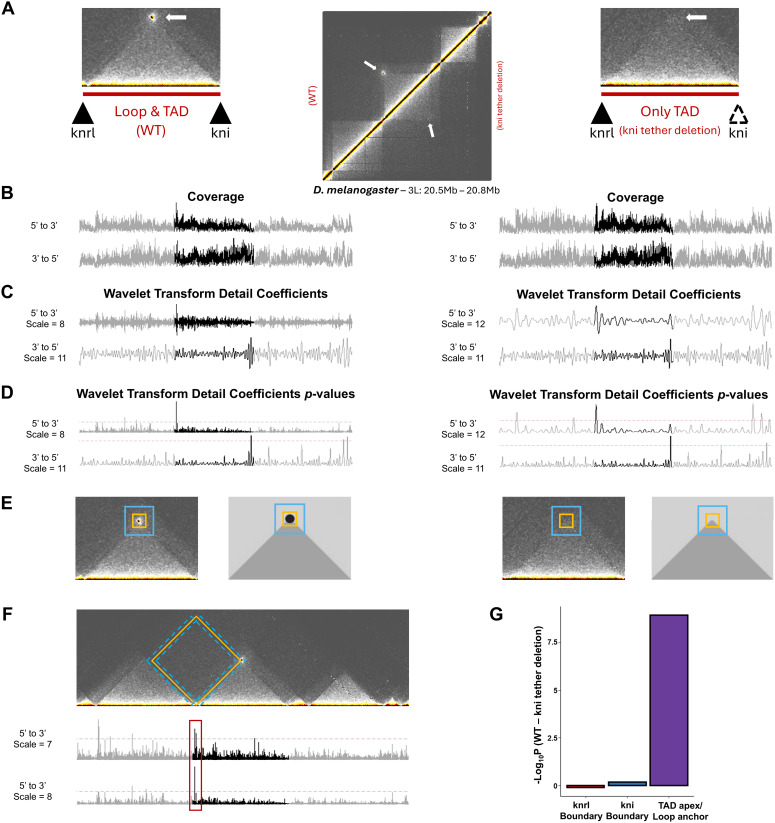
Walkthrough of an application of WaveTAD. **(A)** Contact matrices (1kb resolution) highlighting the loss of the loop structure when the *kni* tethering element is perturbed from Levo et al. [36] **(B)** Underlying coverage signal of the Hi-C matrix for the 5’ (top) and 3’ (bottom) contacts for the wildtype (left) and mutant (right) samples. **(C)** Detail coefficients (shown at two specified scales) generated from the application of wavelet-transforms to the coverage signal. (**D**) *p*-values corresponding to the generated detail coefficients. **(E)** Representative diagram of the “donut” algorithm used to identify both loops and TAD apexes. **(F)** Schematic of the diamond area algorithm overlayed over the control *knrl*-*kni* contact matrix. The diamond area algorithm is used to reconcile scenarios when WaveTAD calls the same TAD boundary at multiple scales with locations slightly moved (red box). **(G)** Boxplot comparing the change in boundary and loop *p*-values (see methods) between the wildtype and perturbed samples. See **Methods** for details.

To highlight the WaveTAD program with a specific case where changes in TAD/loop structures are predicted, we applied it to a pair of Micro-C datasets consisting of a control sample and a sample containing a genetically perturbed tethering element required for a promoter-enhancer interaction [36] (**Fig 2A**). In brief, WaveTAD analyzed the pair-read coverage signal of the samples (**Fig 2B**) to produce detail coefficients across many scales (**Fig 2C**). Locations with significantly elevated detail coefficients (**Fig 2D**) represent areas displaying an enrichment in long-range contacts, a hallmark of TAD and loop boundaries. All possible 5’ - 3’ pairwise contacts (potential TAD/loops) are then generated and the presence of long-distance interactions at their predicted TAD apex/loop anchor locations is investigated with a “donut” algorithm (see **Methods** for details) that assigns an associated probability (**Fig 2E**). Given that wavelet transforms analyze the signal across many scales, leakage across scales when analyzing broad boundaries can generate multiple hits in close proximity to one another. To resolve this issue, WaveTAD applies a diamond area algorithm (see **Methods** for details) relative to the size of the identified TAD (**Fig 2F**).

After the TADs/loops are finally called, we can then compare the TAD boundary and TAD apex/loop anchor strengths between the two samples to gain biological insight. Levo et al. [36] performed a targeted replacement of the tethering element of the *knirps* (*kni*) gene that depletes the contacts between *kni* and its paralogue *knirps-related* (*knrl*). In agreement, our analysis shows reduced apex/anchor strength in mutant compared to wildtype samples, confirming reduced interaction frequency between tethering elements while maintaining internal TAD interactions (**Fig 2G**). Overall, this example demonstrates how the WaveTAD program translates sparse and noisy Hi-C data into discrete TAD/loop, boundary and apex/anchor calls with corresponding strengths that can then be directly compared within a rigorous statistical framework. In a genome-wide context, TAD calls can be compared between samples by using the Jaccard Index (JI) or overlap coefficient. These metrics gauge the similarity between two sample sets and are the current standard in the field for measuring the concordance of TAD calls between samples [15–18].

### WaveTAD identifies TADs independent of resolution, handles sparse matrices, and provides robust and reproducible TAD calling

**Fig 3:**
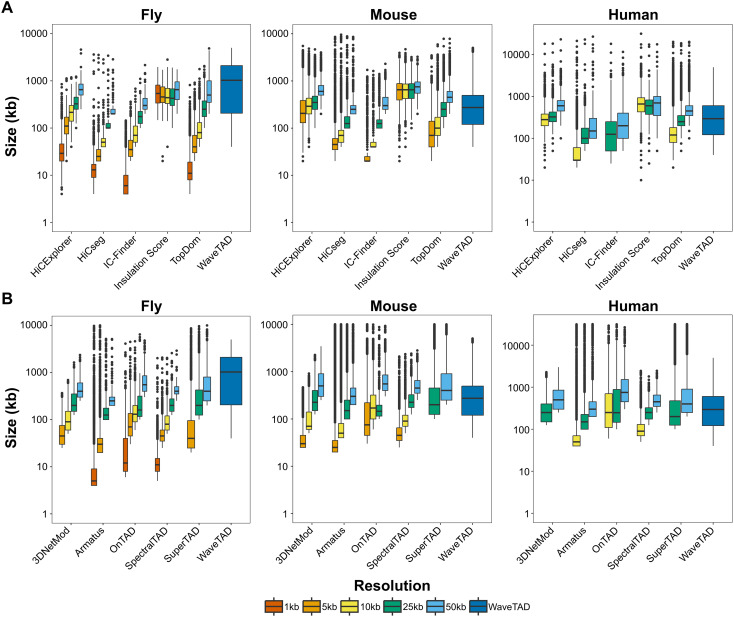
Comparison of TAD calls between methods. Box plots of TAD sizes called by each tool using different resolutions of contact matrices for the different species: fly (1kb, 5kb, 10kb, 25kb, 50kb), mouse (5kb, 10kb, 25kb, 50kb), and humans (10kb, 25kb, 50kb). Note that WaveTAD is resolution-free. (**A**) and (**B**) for non-hierarchical and hierarchical TAD callers, respectively.

To exemplify the importance of a resolution-free TAD caller, fly [37], mouse [38] and human [39] datasets were analyzed by multiple methods at different resolutions (S1**-**S3 Fig). These methods included the non-hierarchical TAD callers HiCExplorer, HiCseq, Insulation Score, IC-Finder, TopDom, and the hierarchical TAD callers 3DNetMod, Armatus, OnTAD, SpectralTAD, and SuperTAD (see **Methods** for details). We first quantified the dependence on resolution by determining the number and size of called TADs (**Fig 3** and S4 Fig). In all datasets, analyses at different resolutions drastically change the detected TADs. Finer resolutions systematically bias towards identifying more and smaller TADs, while broader resolutions reveal fewer, larger TADs. This dependency on resolution is substantial, with the number of TADs and their median size shifting more than an order of magnitude between fine and broad resolutions. On the other hand, WaveTAD calls TADs with a wider range of sizes compared to the other methods, as expected based on the identification of hierarchical 3D structures and its intrinsic resolution-free nature. Moreover, WaveTAD calls equivalent TAD sizes for the three species analyzed. Insulation Score calls a consistent number of TADs regardless of resolution although it calls significantly fewer TADs and with a much narrower range of sizes than WaveTAD. Because resolution in TAD studies is often different for different species and can be also influenced by the number of contact reads in a given experiment, resolution-dependent analyses of TADs could generate inappropriate information about biologically relevant properties (such as differences in average TAD size when comparing species) whereas WaveTAD allows for more direct and objective comparisons.

**Fig 4:**
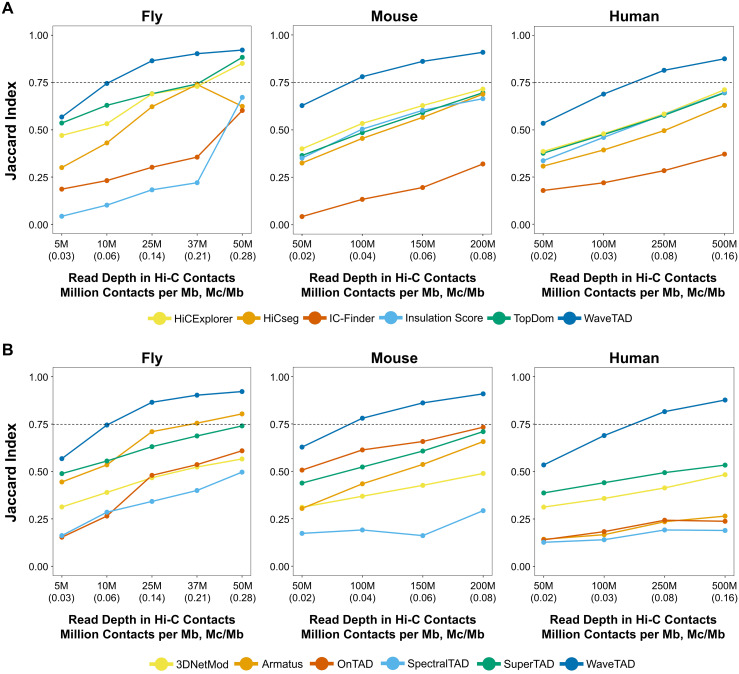
Comparison of TAD calls between methods. Concordance of TAD boundaries using the Jaccard Index across read depths (number of million mapped Hi-C contacts per megabase (Mc/Mb) in parentheses). The Jaccard Index was estimated relative to the highest contact read depth for each species: 75M (0.35 Mc/Mb) for flies, 250M (0.10 Mc/Mb) for mouse, and 1B (0.31 Mc/Mb) for human. A resolution of 10kb for fly and 25kb for mouse and human was used. (**A**) and (**B**) for non-hierarchical and hierarchical TAD callers, respectively.

To explore WaveTAD’s ability to handle sparse data (low number of contact reads per Mb) better than previous methods, we downsampled the fly, mouse, and human datasets and compared the boundaries according to the methodology of benchmarking studies [15,18,40]. For these analyses of sensitivity and accuracy, we analyzed the boundaries called by each tool relative to those identified when using the highest read depth sample for each dataset as reference. Both the Jaccard Index (JI) (**Fig 4**) and the overlap coefficient (S5 Fig) show WaveTAD as the method least influenced by reduced read depths when compared to all other methods in the three species.

True positive rates (TPR) and false discovery rates (FDR) were also determined for each tool by comparing the downsampled reads to the highest read depth sample (S6 Fig). All tools show a reduction in TPR when read depth is reduced, but WaveTAD is the only tool to maintain a high TPR (highest amongst all methods for a given read depth). Importantly, WaveTAD shows high TPR while maintaining a low FDR for each read depth analyzed and across species. Analyses of fly genomes would achieve a TPR above 0.85 and FDR below 0.02 with ~25M contacts (~0.10M contacts per Mb). Analyses of mouse and human genomes would achieve TPR above 0.85 and FDR < 0.05 with ~200M (~0.08 contacts/Mb), and TRP above 0.85 and FDR < 0.06 with ~500M (~0.16 contacts/Mb), respectively. The contact matrices generated for different read depths and the corresponding TADs called by different methods were also plotted as heat maps to allow for visualization (S7**-**S9 Fig).

**Fig 5:**
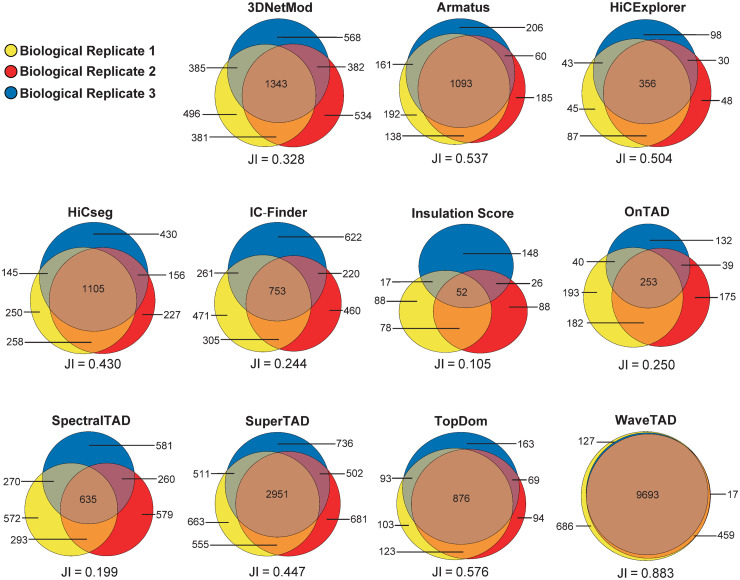
Concordance of TADs called by various tools between biological replicates in *Drosophila melanogaster.* Venn diagram depicting the number of TADs and number of overlapping TADs called between biological replicates. For each tool the Jaccard Index for all replicates is located below the Venn diagram. Data from Hug et al. staged embryos 3-4 hours post fertilization, biological replicates 1-3, (yellow, red, and blue, respectively) [37].

To study the consistency of TAD-calling by the different tools, we analyzed multiple biological and technical replicates from fly datasets (S1 Table) [37] (see **Methods** for details). The concordance (JI) of boundaries called between biological replicates when using WaveTAD was 0.883 whereas it ranged between 0.105 (Insulation Score) and 0.576 (TopDom) for the other methods (**Fig 5**).

Similarly, WaveTAD also had the greatest concordance among all tools to infer TADs between technical replicates (0.825), with SpectralTAD and TopDom generating JI ranging between 0.141 and 0.630, respectively (S10 Fig). WaveTAD also shows a high degree of consistency when calling TAD strengths from biological and technical replicates. When broken up into 25 quantiles, TAD strengths between replicates show a mean Spearman’s ρ of 0.980 and 0.972, for biological and technical replicates, respectively (see S11**-**S12 Fig for results from 10, 25, 50 and 100 quantiles).

### TAD strengths generated by WaveTAD capture TAD frequency in heterogeneous samples

To evaluate the biological relevance of the TAD strengths produced by WaveTAD when analyzing bulk Hi-C data, we compared the frequency of a TAD being called in single-nucleus Hi-C (snHi-C) with TAD strengths generated from bulk analysis from the same genomes. We used snHi-C from mouse embryonic stem cells (mESCs) (S1 Table) [41,42] and selected the top 500 single-nucleus samples in terms of read depth coverage. These snHi-C analyses allowed estimating the frequency a given TAD is called when these 500 samples are analyzed individually. We then generated an *in silico* bulk (‘merged snHi-C’) sample consisting of the first one million reads from each of the top 500 single-nucleus samples (500 million total). We also analyzed a more traditional bulk Hi-C sample from the same mESCs line [42]. TAD strengths from the merged snHi-C and bulk Hi-C datasets were then broken up into multiple quantiles (10, 25, 50 and 100) and compared to the frequency of the TADs called in the snHi-C dataset (S13A Fig). A Spearman's rank order correlation analysis shows very high association between the frequency of TADs from the snHi-C dataset and the strength of the TAD generated by WaveTAD in bulk analyses (either merged snHi-C or bulk Hi-C datasets) (e.g., Spearman’s ρ = 0.968 and 0.945, respectively, when using 25 quantiles). Significant correlations are also observed when restricting the analysis to different TAD size groups (S13B Fig). These results indicate that differences in TAD strength provide information about the variable frequency of these TADs within samples with potentially heterogeneous genomes, thus allowing better inferences about TAD properties and dynamics with bulk Hi-C approaches.

### WaveTAD identifies TADs and boundary changes in heterogeneous samples

**Fig 6:**
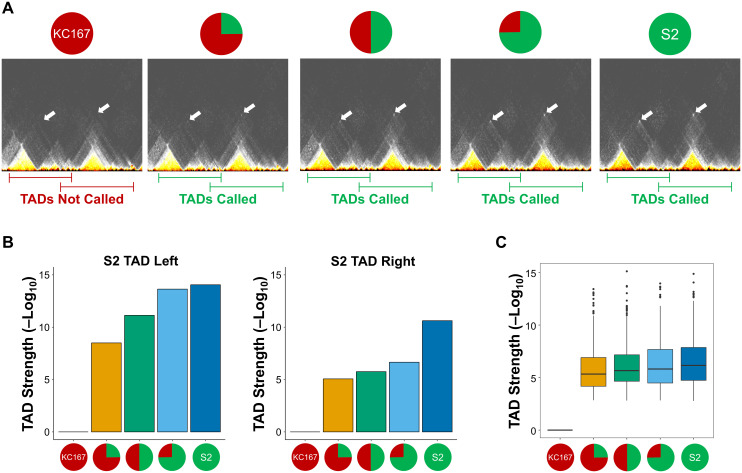
WaveTAD identifies TADs and boundary changes in heterogeneous samples. **(A)** Contact matrices (10kb resolution) containing Hi-C contacts mixed between two *Drosophila melanogaster* cell lines. Shown is a region (3R:22,000,000-23,400,000) with two TADs (white arrows) unique to the S2 cell line (S2 TAD Left and S2 TAD Right). The pie chart above each contact matrix shows the ratio of reads used to build the matrix (1:0, 3:1, 1:1, 1:3, or 0:1 ratio of KC167 to S2, respectively). Below, lines indicate whether WaveTAD called the pair of TADs (green) or not (red). **(B)** Bar plots showing the TAD strength (-Log_10_(*p*)) of each of the two TADs highlighted in **(A)**, across the mixed Hi-C contact samples. **(C)** Genome wide analysis of all TADs unique to the S2 cell line. Bar plot shows median TAD strengths (-Log_10_(*p*)) for the S2-unique TADs given the ratio of Hi-C contacts in the mixed samples (pie charts on the x-axis). For all analyses, contact matrices contained a total of 75 million Hi-C contacts.

To expand on WaveTAD’s sensitivity in calling TADs present only in a fraction of genomes, we analyzed mixed Hi-C data from two samples containing different TAD calls. We used two *Drosophila melanogaster* cell lines (S2 and KC167) (S1 Table) [43,44], and a sample was created to mimic a heterogeneous population of cells by mixing Hi-C contacts from the two cell lines, with either 1:0, 3:1, 1:1, 1:3, or 0:1 ratio of S2 to KC167 reads while maintaining the same total number of usable Hi-C contacts (75M). **Fig 6** shows the analysis of two nearby TADs called in the S2 cell line but not in the KC167 line. Both TADs are identified even when S2 Hi-C reads represent only 25% of the whole sample, with the strength of the TAD call increasing (more statistically significant) with the fraction of genomes containing the TAD (**Fig 6B**). This property remains genome-wide when applied to all S2-specific TADs, with the median strength of these TAD calls increasing as the proportion of the TAD-containing Hi-C reads increases (**Fig 6C**).

We also assessed the ability of the different methods to identify boundaries in heterozygous samples as a function of read depth and resolution in larger genomes. Specifically, we analyzed pluripotent human stem cells (hPSCs) containing a *de novo* human endogenous retrovirus subfamily H (HERV-H) insertion that creates a new TAD boundary [45] (S1 Table). WaveTAD called the newly generated boundary in heterozygous conditions for read depths ranging from 0.1M Hi-C contacts per Mb (6M contact reads) to 0.016M Hi-C contacts per Mb (1M contact reads) (S14 Fig). None of the other methods were consistent in identifying the heterozygous TAD. 3DNetMod, Armatus, HiCExplorer, IC-Finder, SpectralTAD, SuperTAD, and TopDom identified the boundary for some resolutions but not for others. At the lowest read depth, only 3DNetMod, Armatus, SpectralTAD, SuperTAD, and TopDom correctly detected the boundary (and only at specific resolutions), while IC-Finder and SuperTAD called false positives at some resolutions. Insulation Score, HiCseg, and OnTAD did not call the new boundary at any resolution or read depth analyzed.

### Large TADs called by WaveTAD match high-resolution imaging

**Fig 7:**
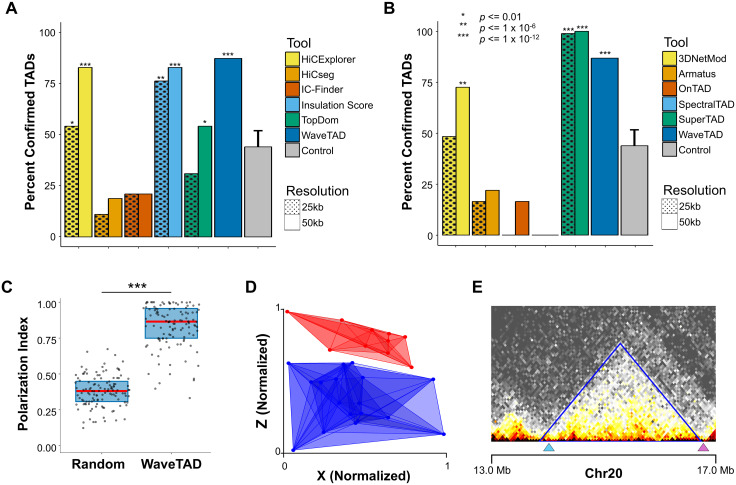
Compartment sized TADs called by WaveTAD match high-resolution imaging. **(A,B)** Percent of FISH-confirmed TADs called by different tools at 25kb and 50kb resolutions (WaveTAD is independent of resolution) (see **Methods** for details). **(C)** Polarization index based on the compartments confirmed by WaveTAD and a randomized control (Wilcoxon Paired Test). Analysis for chromosome 20 from FISH performed on 120 individual cells, where the red line represents the median and the blue boxes represent the 1^st^ and 3^rd^ quartiles. **(D)** Spatial map of the convex hulls for compartment-A (red) and compartment-B TADs (blue). Each TAD position (point) represents the centroid of the 120 individual cells. The spatial map was orientated by rotating the vector connecting the centroid of the convex hulls (A and B compartments) so that it was aligned to the y-axis. **(E)** An example of FISH-validated long-range contacts (blue and purple triangles) presented alongside the corresponding WaveTAD-identified TAD (blue line).

To provide further support to the biological relevance of TADs called by WaveTAD and other TAD callers, we compared predicted TAD boundaries with fluorescence in situ hybridization (FISH)-identified long-range contacts obtained with high-resolution imaging of individual human diploid fibroblast IMR90 cells (S1 Table) [46]. Using bulk Hi-C data from the same study, we analyzed all TADs predicted to be 500kb or longer. Three of the TAD callers identified over 80% of the FISH-identified contacts, with WaveTAD (90.1%) showing the highest percentage, followed by HiCExplorer (83.5%), and Insulation Score (82.4%) (both at 50kb resolution) (**Fig 7A**). At 25kb resolution, the ability of the resolution-dependent tools to call the large TADs greatly deteriorated except for Insulation Score.

Because the genome can be divided into two compartments (A and B) that are associated with open and closed chromatin, with interactions largely constrained within the same compartment, we analyzed whether the large TADs called by WaveTAD showed separation in 3D space based on FISH data [46]. We quantified the polarization index (PI) for TADs to assess overlap (or lack thereof) in 3D space, with PI expected to be 1 when compartments are completely separated in space in a polarized fashion. Large TADs called by WaveTAD are clearly divided into two different partitions, as expected if they define compartments A and B, with a median PI value of 0.865 markedly greater than the randomized control (median value of 0.381) (**Fig 7C**) that can be visually represented by building a spatial position map based on centroid positions for each of these TADs (**Fig 7D**).

### A majority of higher-order chromatin structures appear before zygotic genome activation (ZGA) in both fly and mouse

According to current views in the field, high-order chromatin structures are greatly weakened after fertilization with chromatin in a relaxed state, whereas the majority of the multi-scale 3D chromatin organization emerges alongside with zygote genome activation (ZGA) in early metazoan embryos ([37,47,48]; reviewed in [49–51]). This concept also aligns well with the key role of 3D genomic structures on gene regulation. To investigate whether TADs are indeed uncommon before ZGA or, conversely, previously missed due to weak signals that could represent transient contacts, we studied the early emergence of 3D nuclear hierarchy during fly and mouse development with published Hi-C datasets (S1 Table) [37,47]. The genome of the rapidly dividing embryos of *D. melanogaster* is assumed to be initially unstructured, with a prominent surge in TAD signal occurring during zygotic genome activation (ZGA), which occurs at nuclear cycle (nc) 14 [37]. Similarly, the presence of TADs becomes substantial at the 2-cell stage in mice [47–49].

**Fig 8:**
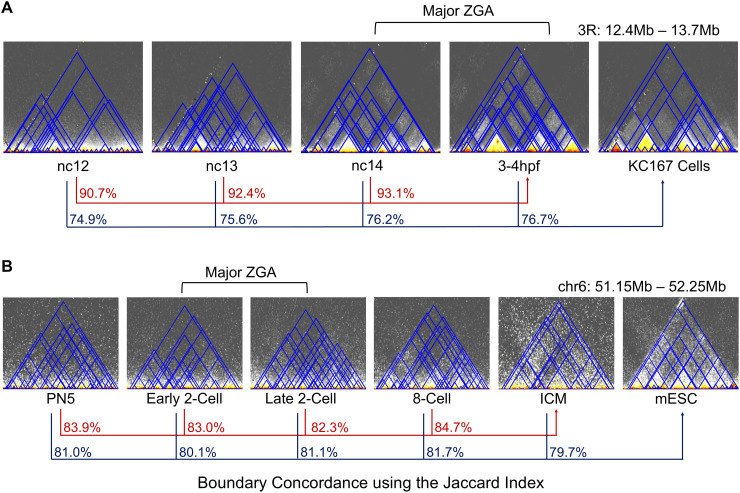
A majority of higher-order chromatin structures appear before zygotic genome activation (ZGA) in both fly and mouse. **(A)** Contact matrices (10kb resolution) of *Drosophila melanogaster* (chr3R:12,400,000-13,600,000) over four developmental timepoints (nc12, nc13, nc14, and 3-4 hours post fertilization, hpf) and the KC167 cell line are overlayed with TADs called by WaveTAD. The red lines and corresponding percentages (in red) show the genome wide concordance of TAD boundaries for each timepoint (nc12, nc13 and nc14) relative to the 3-4 hpf timepoint using the Jaccard Index. The dark blue lines and corresponding percentages show the genome wide concordance of TAD boundaries using the Jaccard Index for each timepoint (nc12, nc13, nc14, and 3-4 hpf, respectively) relative to the KC167 cell line. Note that zygotic genome activation occurs at nc14. **(B)** Contact matrices (10kb resolution) of *Mus musculus* (chr6:51,000,000-53,000,000) over five developmental timepoints (PN5, early 2-cell, late 2-cell, 8-cell, and ICM) and the mESC cell line are overlayed with TADs called by WaveTAD. The red lines and corresponding percentages show the genome wide concordance of TAD boundaries using the Jaccard Index for each timepoint (PN5, early 2-cell, late 2-cell, and 8-cell, respectively) relative to the ICM timepoint. The dark blue lines and corresponding percentages show the concordance of TAD boundaries using the Jaccard Index for each timepoint (PN5, early 2-cell, late 2-cell, 8-cell, and ICM, respectively) relative to the mESC cell line. Note that zygotic genome activation in mouse occurs at the 2-cell stage.

Analysis of the fly datasets using WaveTAD (**Fig 8A**) showed that the great majority of TAD boundaries present after ZGA are also identifiable before ZGA (at nc12), with a boundary concordance (JI) of 90.7%. We further compared early- and mid-stage embryo TADs to KC167 cells, a widely used and well-characterized cell line established from 6-12h embryos with a hemocyte-like mRNA expression pattern [52]. We observed that pre- and post-ZGA samples show similar boundary concordance (JI: 74.9 - 76.7%) when compared to KC167 cells. Similarly, in mice (**Fig 8B**) we observed very high concordance between boundaries called by WaveTAD at the pronuclear stage 3 (22 hours) before ZGA and inner cell masses (ICM) from blastocysts (92–94 hours) after ZGA, with a JI of 0.837. Embryonic mouse samples before and after ZGA show an equivalent high degree of TAD concordance when compared to established mouse embryonic stem cells (mESCs) (JI: 79.7 - 81.7%).

Our analyses using WaveTAD identifies pre-ZGA 3D structures that were previously overlooked likely due to widespread weaker signals, a scenario compatible with pre-ZGA contacts being highly transitory, producing a population of embryonic 3D genomes with TADs at low frequency. To seek support for this possibility and given WaveTAD’s property of generating TAD strengths that capture differences in frequency in heterogeneous populations, we compared the TAD strengths associated with TADs before and after ZGA. In both mouse and fly, TAD strengths increase from before to after ZGA (Mann–Whitney U test *p* < 2.2x10^-16^ in both fly and mouse). Thus, while WaveTAD suggests that most potential 3D genomic structures predate ZGA and are conserved throughout development in both flies and mice, it also identifies pre-ZGA structures as weaker—likely less stable—than those post-ZGA, supporting temporally dynamic pre-ZGA TADs. Recent super-resolution live-cell imaging inferring the existence of very transient structures in mouse embryonic stem cells would support this possibility [53].

### CTCF sites and TAD stability

CTCF is widely regarded as the predominant architectural protein in humans, being responsible for constraining cohesin-mediated loop extrusion via convergently oriented binding sites [54–65]. The 4D Nucleome (4DN) project [66], for instance, assesses the quality of TAD calls based on the overlap with CTCF sites [67–69] and reproducible results across different protocols and sequencing depths. The 4DN project, however, uses Insulation Score to call TADs. Given the difference in sensitivity, specificity, and reproducibility between WaveTAD and Insulation Score shown above, we re-analyzed multiple 4DN datasets of the human embryonic stem cell line H1 (H1-hESC), including standard Hi-C [70], 4DN Tier1 Hi-C [39], and 4DN Tier1 Micro-C [39], where 4DN Tier1 denotes samples with the highest sequencing depth and Micro-C identifies the highest 3D resolution protocol. Out of the reported 69,108 CTCF sites in the ENCODE CHIP dataset (see **Methods** for details), a much larger fraction overlaps with TAD boundaries called by WaveTAD than by Insulation Score, an enrichment that remains significant (Z-score) after considering the higher number of boundaries called by WaveTAD (S15 Fig; see **Methods** for details). These results support the notion that WaveTAD is more sensitive and accurate than Insulation Score at identifying 3D structures.

**Fig 9:**
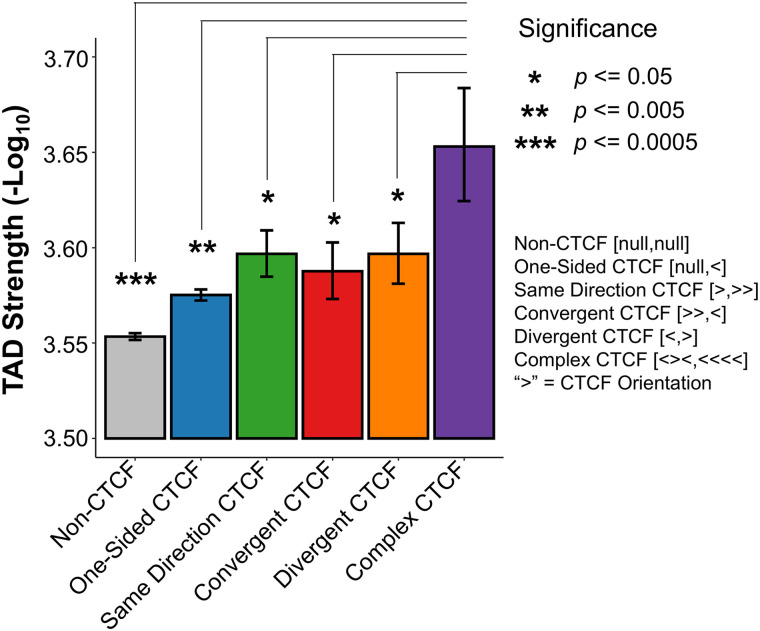
Complex CTCF sites produce more stable TADs. Bar plot showing the average TAD strength (-Log_10_(*p*)) for six classes of TAD boundaries: 1) TADs that contain no CTCF sites at either TAD boundary, 2) TADs with one boundary containing no CTCF sites and the other containing one or more CTCF sites, 3) TADs with both boundaries containing one or more CTCF sites all sharing the same orientation, 4) TADs with both boundaries containing one or more CTCF sites in a convergent orientation, 5) TADs with both boundaries containing one or more CTCF sites in a divergent orientation, and 6) TADs where each boundary contains one or more CTCF sites with at least one of the boundaries showing multiple orientations. The average TAD strengths of each class were compared to the non-CTCF class and *p*-values were generated by bootstrapping.

Taking advantage of the quantitative information provided by WaveTAD, we then studied the impact of number and orientation of CTCF sites on TAD structures (**Fig 9**). For this analysis, we used a collection of 61,079 CTCF sites whose orientations were previously annotated (see **Methods** for details) [54,56]. As expected, TADs with one or more CTCF sites in at least one of the boundaries show increased TAD strengths than TADs without CTCF sites (*p* = 1.06 x 10^-15^), indicating improved stability and higher frequency in the sample. For TAD boundaries with a single CTCF site on each side, there is no difference in estimated strength (*p*-values) for convergent, divergent or same direction CTCF sites (*p* > 0.25 in all 3 pairwise comparisons). TADs with complex CTCF structures at their boundaries (multiple CTCF sites on each side) produce the strongest TADs of any CTCF class. This information supports the previously reported phenomena that TADs are more frequently identified when associated with multiple architectural proteins [43,71–74], and WaveTAD provides statistical support to the increased stability of 3D structures with increased number of CTCF sites from bulk Hi-C data.

### SARS-CoV-2 infection disrupts pathway-specific DNA loops

A common symptom of SARS-CoV-2 infection is anosmia, defined by the partial or complete loss of sense of smell [75–78]. Unlike other upper respiratory infections, COVID-19 mediated olfactory dysfunction does not occur via conductive interference. Instead, a recent Hi-C and RNA-seq study of olfactory epithelium of COVID-19 patients reported downregulation of olfactory receptor (OR) and OR signaling genes together with disrupted inter-chromosomal genomic contacts. The results of this study, therefore, suggest a potential disruption of the nuclear architecture in olfactory sensory neurons as the mechanism by which the virus elicits non-cell-autonomous transcriptional changes in neurons that lack entry receptors [79]. This study, however, only identified a significant reduction in long-range inter-chromosomal contacts (*in trans*) between clusters of olfactory receptor genes, possibly due to an elevated noise-to-signal ratio. We investigated whether WaveTAD could identify additional 3D properties using the same Hi-C and RNA-seq datasets that would provide further links with pathway-specific changes in gene expression regulation [79] (S1 Table).

**Fig 10:**
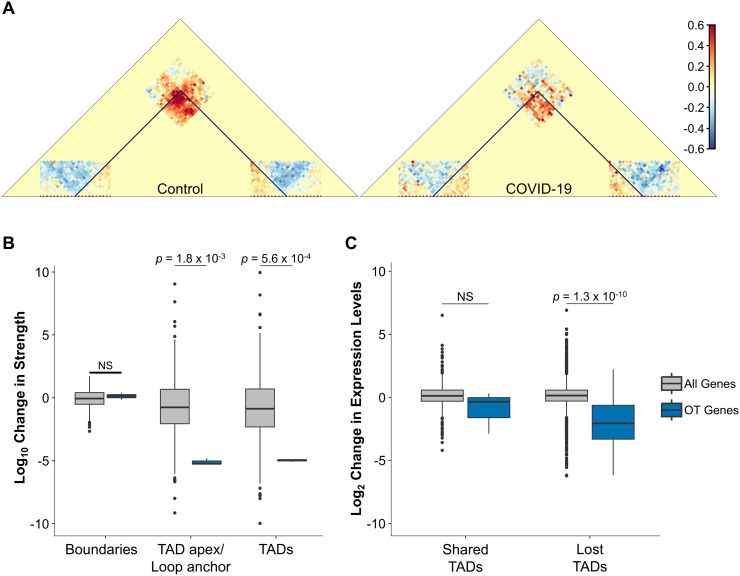
SARS-CoV-2 infection disrupts pathway-specific DNA loops. **(A)** Merged contact matrices of TADs containing olfactory transduction genes at one or both of their boundaries. Contact matrices of TADs were normalized to a randomized set of TADs with similar TAD size to control for the power-law decay observed in Hi-C data. Areas of greater than expected contact densities are shades of red, whereas less than expected contacts densities are shades of blue. **(B)** Box plot of the change (Log_10_) in strength of boundaries, TAD apexes/loop anchors and TADs for TADs/loops that contain at least one olfactory transduction gene (blue). As a comparison, all genome wide TADs that contain at least one gene at their boundaries are also plotted (grey). **(C)** Box plot of the change in gene expression levels (Log_2_-fold) of genes located at TAD boundaries for TADs that are either shared and lost between the control and COVID-19 samples. The box plot is further classified into shared and lost TADs that contain an olfactory transduction gene at one of their boundaries (blue) and TADs that contain any gene at one of their TAD boundaries (grey). All *p*-values were generated by bootstrapping.

Reanalysis of RNA-seq data from olfactory epithelium of COVID-19 infected patients points out the “Olfactory Transduction” (OT) pathway as the most significantly enriched pathway, with downregulation of olfactory receptor signaling genes (S16 Fig). Notably, “Apoptosis” pathway is not significantly enriched, thus ruling out cell death as a cause of differences between control and COVID-19 Hi-C samples. WaveTAD identified significant changes in intra-chromosomal structures. More specifically, this variation is driven by the weakening of chromatin TAD apex/loop anchors rather than boundaries, suggesting more labile promoter-enhancer contacts in COVID-19 patients (**Fig 10A**, **10B**). This notion is further supported by the reduction of expression levels of OT genes located at the boundaries of TADs present in the control but disrupted in COVID-19 patients (**Fig 10C**). These results add an additional nuance to the proposed molecular mechanism of COVID-19-induced anosmia, with the preferential disruption of intra-chromosomal contacts associated with the regulation of OT genes.

### Sex-specific genes and TAD stability in *Drosophila*

Sexual dimorphism can be attributed in large part to the differential expression of genes [80,81]. In *Drosophila* species, sex determination and sex-biased gene expression originate from genetic cascades initiated from the sex chromosomes [82–84]. We took advantage of these sex-specific transcription differences to identify associated changes in 3D architecture. To this end, we used Hi-C datasets from *D. melanogaster* female and male cell lines [43,44] and a list of conserved sex-specific genes across multiple *Drosophila* species [84]. Due to the small number of female-specific genes (see **Methods**), we focused on male-specific genes and potential differences in TAD properties between female and male cell lines.

**Fig 11:**
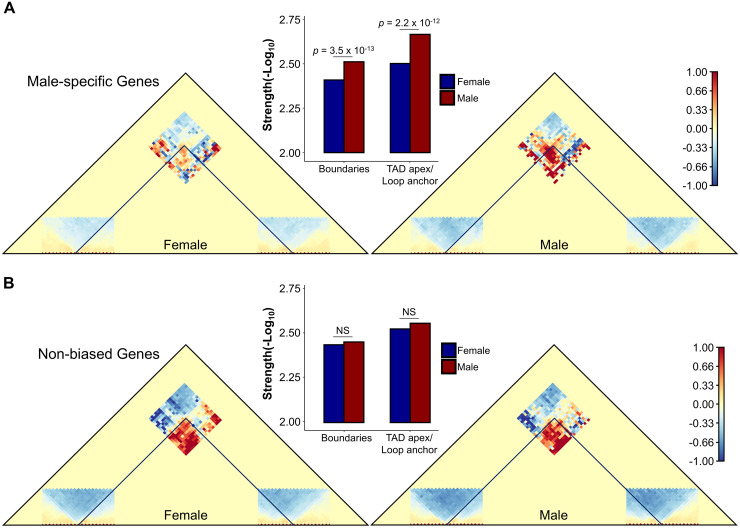
Sex-specific genes and TAD stability in *Drosophila.* **(A)** Merged contact matrices of TADs containing male-specific genes at one or both of their boundaries. Contact matrices of TADs were normalized to a randomized set of TADs with similar TAD size to control for the power-law decay observed in Hi-C data. Areas of greater than expected contact densities are shades of red, whereas less than expected contacts densities are shades of blue. Box plot between male and female contact matrices shows average TAD boundary and loop strengths (-Log_10_(*p*)) of TADs containing male-specific genes for the male and female cell lines (blue and red, respectively). **(B)** Merged contact matrices of TADs containing non-biased genes at one or both of their boundaries. Contact matrices were normalized and depicted as in **(A)**. Box plot between male and female contact matrices shows the average TAD boundary and loop strengths of the TADs containing non-biased genes for the male and female cell lines (blue and red, respectively).

Overall, there is no evidence of changes in TAD presence/absence at genomic locations with male-specific genes when comparing male and female cell lines (*p* > 0.5). There are, however, quantitative differences in TAD structures (**Fig 11A**). 3D structures with male-specific genes show significantly stronger signal at both TAD boundaries and TAD apexes/loop anchors in the male cell line relative to the female cell line, suggesting higher frequency of contacts in the male cell line relative to the female cell. Importantly, there was no statistical difference between the male and female cell lines when comparing TADs with genes known to show similar levels of gene expression in males and females (**Fig 11B**). Overall, this analysis suggests that the association between 3D nuclear structure and transcriptional programs in *D. melanogaster* is quantitative rather than qualitative, even in cases of sex-specific expression, thus explaining previous results suggesting a disconnect between TAD presence and gene expression in *Drosophila* [85].

## Discussion

The accurate annotation of 3D nuclear architecture offers valuable insights. It helps us understand various cellular phenotypes related to gene regulation, development, cancer, and other diseases. Despite significant advances in experimental protocols for both bulk Hi-C and single-cell studies, current analytical frameworks are limited by their resolution-dependent and qualitative nature. These limitations restrict the comprehensive view of multi-layered structures, their configuration, and their dynamic nature. Here, we described WaveTAD, a probabilistic, resolution-free, and hierarchical TAD caller based on WT. Consistent with the other WT applications, WaveTAD is better at identifying 3D structures with sparse signal than previous methods while showing markedly higher sensitivity, specificity, and reproducibility.

A weakness in the benchmarking and validation of TAD callers is the lack of genome-wide gold standard sets of TADs, which should include both genomic location and a measure of stability or frequency in cell populations. While simulated datasets do exist, benchmarking studies emphasize that these algorithms can fail to capture the distribution and biases observed in real Hi-C data [15,40]. To address this lack of a gold standard, we validated WaveTAD, in part, by using TADs that had been corroborated by high-resolution microscopy and genetic means. We further evaluated WaveTAD using the systematic, comprehensive and concise frameworks used by benchmarking studies to assess its accuracy, robustness, and reproducibility. Specifically, we evaluated WaveTAD and other callers across data resolution, sequencing depth, biological replicates, and technical replicates. Furthermore, we validated the use of TAD strengths called by WaveTAD in ‘bulk’ Hi-C analyses as proxy for contact frequency by using snHi-C, demonstrated the ability of WaveTAD to call TADs in heterogeneous samples, and validated large scale TAD calls by using publicly available FISH data. Finally, we demonstrated the broad applicability of this method by using different datasets and model organisms.

We first investigated the resolution dependency of multiple methods including both non-hierarchical and hierarchical TAD callers. Previous work has highlighted the sensitivity of TAD sizes and numbers to changes in resolution [15,18,40]. This can be explained in part by the existence of a hierarchy of TADs, resulting in smaller subTADs becoming more detectable when increasing the resolution of Hi-C matrices [18]. Indeed, the identification of subTADs led to the development of hierarchical TAD callers. However, TAD size and numbers still change dramatically as resolution changes, even for hierarchical TAD callers, with more and smaller TADs identified at high resolution at the expense of their hierarchical larger counterparts (and vice versa at the lower resolutions), likely due to the loss of signal resulting from binning genomic data. WaveTAD alleviates this problem as it analyzes the data at the nucleotide level and automatically identifies the strongest genomic signals at their optimal resolution. As such, it allows for more objective comparisons between studies.

Considering the lack of ground-truth-positive and ground-truth-negative controls, the most common approach to evaluate sensitivity and specificity of TAD callers is to compare performance against downsampled read depths or between replicates. The underlying rationale of the downsampling approach pertains to the idea that increasing sparsity decreases accuracy and reproducibility, thus TAD calls at the highest read depth samples can be used in place of a gold standard set and subsequently used to derive true positive rates and false discovery rates. Highlighting the strengths of wavelet transforms’ ability to handle sparse data, WaveTAD outperformed the other TAD callers. These analyses consistently showed low FDR while maintaining high TPR across a wide range of contact densities in flies, mouse and humans, supporting the conservative nature of our approach to handling variances of detail coefficients across scales for Hi-C datasets without significantly reducing sensitivity.

The benchmarking of TAD callers using measures of concordance between biological or technical replicates is based upon the idea that methods with higher concordance represent higher reproducibility; granted that both biological and technical factors can reduce concordance between replicates, these would affect all methods equally. WaveTAD showed the greatest concordance among all tools to infer TADs between both technical and biological replicates. Note that all TAD callers showed similar concordance between technical and biological replicates, with several, including WaveTAD, estimating slightly higher concordance for biological than for technical replicates, when the opposite was expected [15,40]. These results could be caused by differences in read-depth between replicates, or by true differences between biological replicates due to population of cells being in different states and phases of the cell cycle (as proposed by Forcato et. al. [15]).

The ability of WaveTAD to identify Hi-C signals in noisy and sparse datasets combined with its quantitative approach when calling structures allows detecting the presence of loops and TADs even when they are at low frequency in a sample. This property enables WaveTAD to capture differences in TAD frequency in bulk Hi-C analyses based on the TAD strength associated with each structure. Until now, capturing TAD frequency has been limited to large-scale single-cell Hi-C studies but this comes at the expense of accepting limited genomic resolution. While WaveTAD can also be applied to single-cell datasets (and therefore obtain frequency data directly), our studies show that it can provide both high-resolution and frequency information from heterogeneous bulk Hi-C samples.

The combination of WaveTAD’s properties provides an advantage over current methods and offers a new, more quantitative approach that will be useful to study 3D dynamics and differences in contact frequencies. We also demonstrated the broad applicability of this method by using different datasets and model organisms. For instance, we showed that different CTCF configurations in humans are associated with different TAD strengths, indicating different levels of 3D stability. We also showed that the 3D organization in early embryos is present, albeit in labile form, before ZGA in both mouse and *Drosophila* based on the study of bulk Hi-C. The comparison of TAD strengths also allowed quantifying associations between TAD stability and differential expression in systems as diverse as COVID-19 patients or sex-specific transcription in *Drosophila*.

The biological interpretation of overlapping TADs and loops is not simple nor direct since some boundaries can influence multiple structures. Contrary to algorithms that only consider adjacent structures (and, therefore, can oversimplify the 3D genome), methodologies that infer hierarchical structures (including, but not solely, WaveTAD) are a step in the right direction. WaveTAD generates additional information by providing matched border-apex-border strength estimates, which can allow studying the different components separately. By providing strength values for TADs and their components, WaveTAD will also allow prioritization and filtering of structures for experimental validation or manipulation.

## Methods

The WaveTAD pipeline (**Fig 1**) begins by mapping paired-end Hi-C sequencing reads using BWA-MEM [86] with the parameters -E 50 -L 0 [87] to the reference genomes of *D. melanogaster* (dm6), mouse (mm10) and human (hg38). Contact matrices were built using HiCExplorer at 1kb (flies only), 5kb, 10kb, 25kb, and 50kb resolutions. These contact matrices were then corrected using HiCExplorer’s hicCorrectMatrix with default parameters and normalized using HiCExplorer’s hicNormalize [87]. When necessary for a TAD caller, the cooler dump utility was used to convert the contact matrices into contact tables [88].

For WaveTAD, mapped reads were filtered to only include read pairs having intrachromosomal interactions, a minimum insert length of 500 bp, and a maximum insert length of 5Mb. After filtering, pairs are split into 5’ and 3’ groups (defining the direction of the interaction in linear space; e.g., 5’ reads interact with their paired read located 3’ along the chromosome). The coverage, in the form of a per-base report, was then calculated for the 5’ and 3’ groups using bedtools genomecov (parameters: -d) [89]. Regions with zero coverage depth were removed and the coverage was subsequently log transformed.

### TAD boundary calling with wavelet transforms

The 5’ and 3’ read depth coverages were used as signal and subjected to a WT algorithm to identify TAD boundaries using Maximum Overlap Discrete Wavelet-Transform (MODWT) with the coiflet filter (c6) from the R package ‘wavelets’ [90]. MODWT is different and has several advantages over Discrete Wavelet Transform (DWT) in that it improves the alignment of the detail (or wavelet) coefficients with the original signal and has no boundary effects, helping with the analysis of signal variation with respect to scale and location [23]. After filtering (convolution) and multi-level decomposition, detail coefficients at each level of decomposition and location across the genome are given by


$$\begin{array}{c} {\tilde{W}}_{j,t}\equiv \;\sum_{l\;=\;\text{0}}^{L_{j}\;-\;\text{1}}{\tilde{h}}_{j,l}X_{t-\text{1}\;mod\;N} \end{array}$$ (1)

where j is the scale, t is the location, l  is the length index, ${\tilde{h}}_{j,l}$ is the coiflet filter, and $X_{t-\text{1}\;mod\;N}$ is the circulatory filter [23]. Assuming independence between data values, the expected variance of the detail coefficients at a given scale decreases by a factor of 2 for each increasing scale, and can be estimated from the variance at the first scale [23,91,92]. The rationale is that the finest scale in the wavelet transform primarily captures high-frequency noise that can be used to infer noise across higher scales, while the signal information is typically spread across coarser scales [93]. In genomic analyses, however, the assumption of independence is unlikely to be correct, particularly at smaller scales. To be conservative, analyses at scale *j* assumed the variance of the previous, smaller, *j* -1 scale. Finally, the asymptotic distribution of the variance at a given scale is asymptotically normal with a mean of 0, which allows the formulation of confidence intervals [23,91,92]. The *p*-values were then adjusted for multiple comparisons using the Holm method. Adjusted *p*-values were filtered using an α = 0.05. At locations where neighboring base pairs also passed this threshold, the mean location of the run of significant base pairs was used. Overall, the result is two lists of high-value detail coefficients and corresponding probabilities that allow identifying locations with a significant increase in contact frequency along a chromosome, defining TAD and loop 5’ and 3’ boundaries.

After assigning probabilities to boundaries, the potential 5’ and 3’ boundaries were paired to form potential TADs or loops. Potential TADs and loops smaller than a minimum size (40kb in both flies and mammals [1]) and larger than 5Mb (more lenient than the proposed 1Mb in flies [73] and 3Mb in mammals [1]) were removed. TADs and loops were then selected based on the presence of a loop anchor or a prominent TAD apex as determined by HiCExplorer’s hicDetectLoops algorithm [94], which incorporates a “donut” layout proposed by HiCCUPS [56,95]. This “donut” algorithm compares the contact frequency of a central (focal) pixel to a local background region around the focal point (“donut” or annulus) and was applied only at locations of potential contacts between boundaries identified by WaveTAD. Because HiCCUPS is not resolution-independent, we analyzed binned matrices at multiple resolutions with relaxed parameters (-p 1 -pw 2 -w 5 -pp 0.1 -pit 10 -oet 1.5 --maxLoopDistance 5000000), which allowed for overlapping calls between resolutions. Additionally, the relaxation of parameters allows identifying both loop anchors and TAD apexes. In cases where the “donut” algorithm generates significant calls at multiple resolutions, the minimum *p*-value is assigned. Importantly, shared calls made by the “donut” algorithm between different contact matrix resolutions produced highly correlated *p*-values (e.g., Pearson’s correlation *p* < 1 × 10^-150^ in genome-wide analyses of *Drosophila* 3–4 hours post fertilization (hpf) between 5- and 10kb, 10- and 25kb and 25- and 50-kb resolutions). If the coordinates of a proposed TAD apex or loop anchor failed to overlap with a “donut” algorithm call, it was removed.

The final filtering is applied to fine-tune the location of broad TAD boundaries when WaveTAD identifies adjacent signals across multiple scales (**Fig 2F**). To resolve these cases, a diamond area algorithm is applied. Some TAD callers use a variation of the algorithm first used by TopDom to call TADs [87,96,97], while others use a similar approach by comparing the contact density within a proposed TAD to the surrounding contact density [98–101]. Based on the size of the proposed TAD being called by WaveTAD, we apply the TopDom algorithm at different resolutions to fine-tune the location of these broad TAD boundaries. In these scenarios, the boundary location and *p*-value are assigned based on the scale that generates the minimum *p-*value.

Finally, we assign a measure of strength to each TAD based on the product of the probabilities for both boundaries derived by WaveTAD (see above) and the probability of the TAD apex/loop anchor from the “donut” algorithm (see above). Considering WaveTAD is designed to identify areas of high contact density, any multiscale structure fitting this definition is identified, including loops, TADs and subTADs, corner-dot TADs, compartments, etc. Thus, for simplicity, WaveTAD “TAD calls” broadly encompass a range of multiscale structures.

### Parameters of the different TAD calling methods used for comparison

The 3D tools chosen for comparison were selected based on their diversity of approach, popularity, and third-party assessments [15–18]. The algorithmic approaches included network features (3DNetMod [102]), linear score (Armatus [103], HiCExplorer [87], Insulation Score [104], OnTAD [96], SpectralTAD [105], SuperTAD [106], and TopDom [97]), statistical model (HiCseg [107]) and clustering (IC-Finder [108]) [16]. Using the number of citations as the criterion for popularity, each tool is among the top for each of the algorithm classes. For all tools, the default user-defined parameters were used except where deviations were recommended by the authors or other published materials (see S2 Table for details).

### Computational resources

All TAD calling methods were run on the University of Iowa Argon High Performance Computing Cluster. Specifically, the methods were run on an Intel 128-core Xeon 6430 2.1GHz (turbo up to 2.6GHz) CPU with 512GB of memory. S2 Table shows runtime and peak memory information for the different methods when calling TADs for human chromosome I with 1 billion valid Hi-C read pairs.

### Comparing TAD boundaries between samples

The Jaccard Index (JI) and the overlap coefficient were used as metrics to measure concordance between samples, conditions or methods. TAD boundaries were considered the same between two sets if they were within one bin of each other on either side. For WaveTAD, which calls a boundary at a single nucleotide location rather than a bin, the spatial tolerance on each side was matched to the bin size leeway of the other tools. When the bin size is large, a single boundary could be concordant with more than one boundary of the other sample. Thus, the intersection of A and B (where A is the set of TAD boundaries in one sample and B is the set of TAD boundaries in another sample) may be different from the intersection of B and A, which leads to a slight modification to the Jaccard Index and overlap coefficient equations. The Jaccard Index was defined as follows:


$$\begin{array}{c} \text{JI}(A,B)=\;\frac{mean(|A\cap B|,\;|B\cap A|)}{\left|A\cup B\right|} \end{array}$$ (2)

Where JI is the Jaccard Index, A is the set of TAD boundaries in one sample and B is the set of TAD boundaries in another sample. While the overlap coefficient was defined as follows:


$$\begin{array}{c} oc(A,B)=\;\frac{min(|A\cap B|,\;|B\cap A|)}{min(|A|,|B|)} \end{array}$$ (3)

Where oc is the overlap coefficient, A is the set of TAD boundaries in one sample and B is the set of TAD boundaries in another sample.

### Determining true positive rate and false discovery rate

As a statistical measure of performance, true positive rates (TPR) and false discovery rates (FDR) were calculated. These metrics were used to evaluate tool performance across read depths. To do so, true positives (TP) were defined as TAD boundaries called in the highest read depth sample and in the downsampled read depth sample. False negatives (FN) were defined as TAD boundaries not called in a downsampled sample that were called in the highest read depth sample. False positives (FP) were defined as TAD boundaries that were called in a downsampled sample but not called in the highest read depth sample. Consistent with the calculation of concordance, a cushion of one bin on each side of a called TAD boundary was implemented when determining whether calls matched, with WaveTAD being assigned a comparable window based on the resolution used by the other tools. Based on these definitions, TPR and FDR were calculated using the following equations:


$$\begin{array}{c} TPR=\;\frac{TP}{TP+FN} \end{array}$$ (4)

$$\begin{array}{c} FDR=\;\frac{FP}{FP+TP} \end{array}$$ (5)

### Comparing single-nucleus Hi-C data to merged and bulk Hi-C data

The single-nucleus Hi-C dataset from mouse embryonic stem cells (see S1 Table) was demultiplexed following the scHiCExplorer documentation and using the scHicDemultiplex algorithm [41,94]. The single-nucleus Hi-C dataset was then mapped, processed and analyzed by WaveTAD in the same fashion as bulk Hi-C. For the single-nucleus Hi-C dataset, only the top 500 single-nucleus samples in terms of read depth coverage were chosen and the TAD calls were combined into a single file. For the ‘merged snHi-C’ dataset, the first one million reads from each fastq file of the same 500 single-nucleus samples were appended to create the two split-read fastq files that were then treated as a bulk Hi-C sample in terms of mapping, processing and WaveTAD analysis. The ‘bulk Hi-C’ dataset represents a multi-cell Hi-C experiment using the same cell line as the single-nucleus Hi-C dataset [41,42]. After calling all TADs for the snHi-C, merged snHi-C, bulk Hi-C and datasets, overlapping TAD calls were generated using a 25kb leeway and correlated to each other. When comparing the samples to each other, only the intersection of the sets that had an assigned probability or nonzero frequency were compared. The merged and bulk TAD strengths were then broken down into quantiles and correlated to the inverse mean TAD frequency for each quantile from the single-nucleus Hi-C data.

### Identifying heterogeneous TADs and TAD boundaries

To mimic a heterogeneous population of cells, the *Drosophila melanogaster* cell lines S2 [44] and KC167 [43] were downsampled to 75 million valid Hi-C contacts and processed using the WaveTAD pipeline. TADs present in the S2 sample and absent in the KC167 sample were then identified. Mixed samples were created by mixing various ratios (3:1, 1:1, or 1:3 ratios) of KC167 and S2 contacts. TADs in the mixed samples were considered concordant to the S2 unique TADs if their respective boundaries were within 50kb.

The HERV-H insertion, and corresponding wildtype Hi-C datasets were downloaded, processed, and analyzed by the different methods to produce all TAD calls for the human chromosome 20. The precise location of the HERV-H insertion was previously documented and converted from its hg19 reference location to its hg38 counterpart. A tool’s TAD boundary call was considered a correct HERV-H insertion boundary call (true positive) if the insertion site was at most one neighboring bin away from the insertion site. A tool’s TAD boundary call was considered a false positive boundary call if they called the insertion site (under the same criteria) in the wildtype sample. This process was expanded to varying read depths (1 million, 2 million, and 3 million).

### Comparing high-resolution imaging of TADs to Hi-C derived TADs

We used high-resolution images of 91 compartment-sized TADs from human diploid fibroblast IMR90 cells [46,109] (S1 Table). These FISH images were obtained after designing probes to bind within the individual compartments [46]. Subsequent analysis validated that A and B compartments are spatially organized and that the spatial distance was correlated to the inverse Hi-C frequency from these same IMR90 cells. We used the IMR90 raw Hi-C dataset [109] and mapped, processed, and analyzed it by the different TAD-calling tools. To determine the accuracy of each tool to call compartment-sized, FISH-confirmed TADs, all TADs under 500kb in size were first filtered out from the individual tool calls. The remaining compartment-sized TADs were then compared to the midpoint of the FISH-confirmed TADs. If they overlapped with the midpoint, then the conclusion was that the called TADs overlapped with at least 50% of the image-verified TAD and was confirmed. The percentage of confirmed Hi-C TADs was compared to that of a control set. This control set was obtained by randomly generating 10,000 sets of size-controlled and non-overlapping TADs, and comparing their locations to the 91 FISH validated TADs. Probabilities and confidence intervals were obtained based on the distribution of percentage of confirmed TADs from the 10,000 control sets.

The polarization index was calculated for each FISH replicate of chromosome 21 based on the previously described compartment assignments and the following equation [46]:


$$\begin{array}{c} Polarization\;Index=\;\sqrt{\frac{\text{1}-\frac{Vs}{Va}}{\text{1}-\frac{Vs}{Vb}}} \end{array}$$ (6)

Where Va is the convex hull volume of compartment A TADs, Vb is the convex hull volume of compartment B TADs, and Vs is the shared volume.

To create the spatial map of the compartments, we calculated the centroid position of all the individual TADs for the 120 replicates. The A and B compartment assignments were based on the correlations between the PCA and chromatin state described in [46]. We then calculated the convex hull for each compartment and their centroid position and defined the vector between each compartment’s centroid as the polarization axis. The compartments were then rotated so that the polarization axis was in the z direction.

### CTCF and TAD properties

To investigate the association between TADs and CTCF presence we took advantage of human Embryonic Stem Cell (hESC) line H1 Hi-C and Micro-C datasets [39,70] used in the 4D Nucleome (4DN; https://4dnucleome.org/) project [66]. Following the 4DN guidelines we used the CTCF sites generated from the ENCODE CTCF ChIP-seq Replicate 1 dataset (ENCFF692RPA) [68,69], with a total of 69,108 CTCF sites reported for H1-hESC, and compared CTCF presence and boundary calls using Insulation Score (as in the 4DN project) and WaveTAD. Note that WaveTAD calls more TAD boundaries (Hi-C = 101,368, 4DN Tier1 Hi-C = 110,482, 4DN Tier1 Micro-C = 132,273) than Insulation Score (Hi-C = 10,810, 4DN Tier1 Hi-C = 9,495, 4DN Tier1 Micro-C = 11,793) and, therefore, enrichment was measured by Z-scores derived by bootstrapping. For comparing the impact of CTCF orientation and complexity on TAD strength, we used previously defined CTCF orientations from Nanni et. al. [54] for the 61,709 CTCF sites reported by Rao et. al. [56] and calculated *p*-values using bootstrapping.

### Consistency of TAD-calling by different methods

We analyzed multiple technical and biological Hi-C replicates from *Drosophila melanogaster* embryos 3–4 hpf [37] (S1 Table) and estimated boundary concordance using the Jaccard Index (JI; see above). Note that each technical replicate contained between 29 million and 31 million valid Hi-C contacts, while the biological replicates varied between 64 million and 95 million valid Hi-C contacts. These discrepancies in read depth likely caused some of the tools, including WaveTAD, to perform slightly better (in terms of JI) when analyzing the biological replicates rather than the technical replicates.

### Analysis of TAD structures in early fly and mouse embryogenesis

*Drosophila melanogaster* embryonic Hi-C datasets across four timepoints in development (nc12, nc13, nc14, 3–4hpf) were obtained from [37] (S1 Table). For each biological replicate, all technical replicates were combined. The result was three biological replicates for each timepoint, consisting of at least 63 million valid Hi-C contacts. The Hi-C data were then processed and analyzed with WaveTAD. Estimates of boundary concordance using the Jaccard Index (JI; see above) for the fly dataset are the average JI from all possible pairwise comparisons between biological replicates of the nc12, nc13, or nc14 timepoints with the three replicates of the 3–4hpf timepoint. All timepoint biological replicates were also compared to two biological replicates of the KC167 cell line, consisting of ~50 million and ~87 million valid Hi-C contacts [43].

Mouse embryonic Hi-C datasets across five timepoints in development (PN5, early 2-cell, late 2-cell, 8-cell, and ICM) were obtained from [48] (S1 Table). All technical and biological replicates were combined to reach sufficient read depth. The result was one Hi-C sample for each timepoint consisting of at least 265 million valid Hi-C contacts. The Hi-C data was then processed and analyzed with WaveTAD. Estimates of boundary concordance using the Jaccard Index (JI; see above) for the mouse dataset are the average JI from pairwise comparisons between the PN5, early 2-cell, late 2-cell, or 8-cell timepoints with the ICM timepoint. All timepoints were also compared to the mESC cell line consisting of 250 million valid Hi-C contacts [38].

### Gene expression pathways in COVID-19 patients

RNA-seq (tsv files) and Hi-C datasets (pairs files) were downloaded from the 4D Nucleome Data Portal (S1 Table) [66,79]. The RNA-seq datasets were analyzed using DESeq2 [110] and subsequently used to generate a Z-score expression heatmap for olfactory transduction genes derived from a KEGG analysis. The KEGG analysis was performed using KEGGREST [111] and *p*-values obtained by a Mann-Whitney test. Hi-C pairs files were converted to sam files and analyzed by WaveTAD. Merged contact matrices were created by merging TADs (with standardized TAD sizes) that contained an olfactory transduction gene at one or both of its boundaries. A gene was considered overlapping if it was within 10kb of a TAD boundary. Shared TADs between control and COVID-19 samples were identified by requiring both boundaries to be within 10kb from each other. All *p*-values were generated by bootstrapping.

### Sex-specific genes and TAD stability in *Drosophila*

WaveTAD was used to identify TADs in *D. melanogaster* Hi-C datasets derived from male (S2) and female (KC167) cell lines [43,44]. These TADs were then filtered based on whether a gene was within 10kb of either of its boundaries. The genes were further annotated using a previously curated list of conserved sex-biased genes across multiple *Drosophila* species [84]. Because the number of female-specific genes (130 genes) is much smaller than that of male-specific genes (1,313 genes), our analyses of TAD presence and properties focused on male-specific and non-biased genes. Merged contact matrices for male-specific genes were created by merging TADs (TAD size was standardized) that contained a male-specific gene at one or both of its boundaries in either the male or female cell line. This set of TADs was then normalized to a control set of randomized TADs. This process was repeated to create merged contact matrices for TADs containing non-biased genes. To determine whether the TAD structures composing the TADs containing male-specific genes were consistently stronger in the male cell line relative to the female cell line, a sign test was performed. As a control, this test was repeated for the non-biased genes.

## Supporting information

S1 Table Datasets used in this study and corresponding accession numbers. (XLSX)

S2 Table Parameters, total runtime and memory needs when calling TADs on human chromosome 1 with different methods. (XLSX)

S1 Fig TADs called by various TAD callers across resolutions for *Drosophila melanogaster.* (PDF)

S2 Fig TADs called by various TAD callers across resolutions for *Mus musculus.* (PDF)

S3 Fig TADs called by various TAD callers across resolutions for *Homo sapiens.* (PDF)

S4 Fig Number of TAD and TAD boundary calls by various TAD callers across resolutions. (PDF)

S5 Fig Comparison of TAD calls between methods. (PDF)

S6 Fig True positive (TPR) and false discovery (FDR) rates of TAD boundaries called by various tools across read depths. (PDF)

S7 Fig TADs called by various TAD callers across read depths for *Drosophila melanogaster.* (PDF)

S8 Fig TADs called by various TAD callers across read depths for *Mus musculus.* (PDF)

S9 Fig TADs called by various TAD callers across read depths for *Homo sapiens.* (PDF)

S10 Fig Concordance of TADs called by various tools between technical replicates in *Drosophila melanogaster.* (PDF)

S11 Fig WaveTAD probabilities are reproducible between biological replicates. (PDF)

S12 Fig WaveTAD probabilities are reproducible between technical replicates. (PDF)

S13 Fig TAD strengths generated by WaveTAD capture TAD frequency in heterogeneous samples. (PDF)

S14 Fig WaveTAD calls TAD boundaries in cells with heterozygous alleles at low read depths. (PDF)

S15 Fig CTCF sites and TAD stability. (PDF)

S16 Fig SARS-CoV-2 infection downregulates olfactory transduction genes. (PDF)

## References

[1] Bonev B, Cavalli G. Organization and function of the 3D genome. Nat Rev Genet. 2016;17(11):661–78. doi: 10.1038/nrg.2016.112 27739532

[2] Szabo Q, Bantignies F, Cavalli G. Principles of genome folding into topologically associating domains. Sci Adv. 2019;5(4):eaaw1668. doi: 10.1126/sciadv.aaw1668 30989119 PMC6457944

[3] Oldridge DA, Wood AC, Weichert-Leahey N, Crimmins I, Sussman R, Winter C, et al. Genetic predisposition to neuroblastoma mediated by a LMO1 super-enhancer polymorphism. Nature. 2015;528(7582):418–21. doi: 10.1038/nature15540 26560027 PMC4775078

[4] Flavahan WA, Drier Y, Liau BB, Gillespie SM, Venteicher AS, Stemmer-Rachamimov AO, et al. Insulator dysfunction and oncogene activation in IDH mutant gliomas. Nature. 2016;529(7584):110–4. doi: 10.1038/nature16490 26700815 PMC4831574

[5] Ji X, Dadon DB, Powell BE, Fan ZP, Borges-Rivera D, Shachar S, et al. 3D chromosome regulatory landscape of human pluripotent cells. Cell Stem Cell. 2016;18(2):262–75. doi: 10.1016/j.stem.2015.11.007 26686465 PMC4848748

[6] Katainen R, Dave K, Pitkänen E, Palin K, Kivioja T, Välimäki N, et al. CTCF/cohesin-binding sites are frequently mutated in cancer. Nat Genet. 2015;47(7):818–21. doi: 10.1038/ng.3335 26053496

[7] Ibn-Salem J, Köhler S, Love MI, Chung H-R, Huang N, Hurles ME, et al. Deletions of chromosomal regulatory boundaries are associated with congenital disease. Genome Biol. 2014;15(9):423. doi: 10.1186/s13059-014-0423-1 25315429 PMC4180961

[8] Valton A-L, Dekker J. TAD disruption as oncogenic driver. Curr Opin Genet Dev. 2016;36:34–40. doi: 10.1016/j.gde.2016.03.008 27111891 PMC4880504

[9] de Laat W, Duboule D. Topology of mammalian developmental enhancers and their regulatory landscapes. Nature. 2013;502(7472):499–506. doi: 10.1038/nature12753 24153303

[10] Williamson I, Kane L, Devenney PS, Flyamer IM, Anderson E, Kilanowski F, et al. Developmentally regulated Shh expression is robust to TAD perturbations. Development. 2019;146(19):dev179523. doi: 10.1242/dev.179523 31511252 PMC7212092

[11] Jerkovic I, Cavalli G. Understanding 3D genome organization by multidisciplinary methods. Nat Rev Mol Cell Biol. 2021;22(8):511–28. doi: 10.1038/s41580-021-00362-w 33953379

[12] Galitsyna AA, Gelfand MS. Single-cell Hi-C data analysis: safety in numbers. Brief Bioinform. 2021;22(6):bbab316. doi: 10.1093/bib/bbab316 34406348 PMC8575028

[13] Belaghzal H, Dekker J, Gibcus JH. Hi-C 2.0: an optimized Hi-C procedure for high-resolution genome-wide mapping of chromosome conformation. Methods. 2017;123:56–65. doi: 10.1016/j.ymeth.2017.04.004 28435001 PMC5522765

[14] Schwartz YB, Cavalli G. Three-dimensional genome organization and function in drosophila. Genetics. 2017;205(1):5–24. doi: 10.1534/genetics.115.185132 28049701 PMC5223523

[15] Forcato M, Nicoletti C, Pal K, Livi CM, Ferrari F, Bicciato S. Comparison of computational methods for Hi-C data analysis. Nat Methods. 2017;14(7):679–85. doi: 10.1038/nmeth.4325 28604721 PMC5493985

[16] Zufferey M, Tavernari D, Oricchio E, Ciriello G. Comparison of computational methods for the identification of topologically associating domains. Genome Biol. 2018;19(1):217. doi: 10.1186/s13059-018-1596-9 30526631 PMC6288901

[17] Dali R, Blanchette M. A critical assessment of topologically associating domain prediction tools. Nucleic Acids Res. 2017;45(6):2994–3005. doi: 10.1093/nar/gkx145 28334773 PMC5389712

[18] Xu J, Xu X, Huang D, Luo Y, Lin L, Bai X, et al. A comprehensive benchmarking with interpretation and operational guidance for the hierarchy of topologically associating domains. Nat Commun. 2024;15(1):4376. doi: 10.1038/s41467-024-48593-7 38782890 PMC11116433

[19] Harris HL, Gu H, Olshansky M, Wang A, Farabella I, Eliaz Y, et al. Chromatin alternates between A and B compartments at kilobase scale for subgenic organization. Nat Commun. 2023;14(1):3303. doi: 10.1038/s41467-023-38429-1 37280210 PMC10244318

[20] Goel VY, Huseyin MK, Hansen AS. Region capture micro-C reveals coalescence of enhancers and promoters into nested microcompartments. Nat Genet. 2023;55(6):1048–56. doi: 10.1038/s41588-023-01391-1 37157000 PMC10424778

[21] Kim H-J, Yardımcı GG, Bonora G, Ramani V, Liu J, Qiu R, et al. Capturing cell type-specific chromatin compartment patterns by applying topic modeling to single-cell Hi-C data. PLoS Comput Biol. 2020;16(9):e1008173. doi: 10.1371/journal.pcbi.1008173 32946435 PMC7526900

[22] Ramani V, Deng X, Qiu R, Gunderson KL, Steemers FJ, Disteche CM, et al. Massively multiplex single-cell Hi-C. Nat Methods. 2017;14(3):263–6. doi: 10.1038/nmeth.4155 28135255 PMC5330809

[23] Percival DB, Walden AT. Wavelet methods for time series analysis. Cambridge University Press; 2006.

[24] Unser M. Wavelets: on the virtues and applications of the mathematical microscope. J Microsc. 2014;255(3):123–7. doi: 10.1111/jmi.12151 25040167

[25] Akay M. Wavelets in biomedical engineering. Ann Biomed Eng. 1995;23(5):531–42. doi: 10.1007/BF02584453 7503456

[26] Wang JZ. Wavelets and imaging informatics: a review of the literature. J Biomed Inform. 2001;34(2):129–41. doi: 10.1006/jbin.2001.1010 11515412

[27] Denault WRP, Gjessing HK, Juodakis J, Jacobsson B, Jugessur A. Wavelet screening: a novel approach to analyzing GWAS data. BMC Bioinformatics. 2021;22(1):484. doi: 10.1186/s12859-021-04356-5 34620077 PMC8499521

[28] Ben-Yaacov E, Eldar YC. A fast and flexible method for the segmentation of aCGH data. Bioinformatics. 2008;24(16):i139-45. doi: 10.1093/bioinformatics/btn272 18689815

[29] Lee W, Morris JS. Identification of differentially methylated loci using wavelet-based functional mixed models. Bioinformatics. 2016;32(5):664–72. doi: 10.1093/bioinformatics/btv659 26559505 PMC4907398

[30] Abuhamdia T, Taheri S. Wavelets as a tool for systems analysis and control. Journal of Vibration and Control. 2015;23(9):1377–416. doi: 10.1177/1077546315620923

[31] Mulcahy C. Plotting and scheming with wavelets. Mathem Magaz. 1996;69(5):323–43. doi: 10.1080/0025570x.1996.11996470

[32] Addison PS. Wavelet transforms and the ECG: a review. Physiol Meas. 2005;26(5):R155-99. doi: 10.1088/0967-3334/26/5/R01 16088052

[33] Kvikstad EM, Chiaromonte F, Makova KD. Ride the wavelet: a multiscale analysis of genomic contexts flanking small insertions and deletions. Genome Res. 2009;19(7):1153–64. doi: 10.1101/gr.088922.108 19502380 PMC2704434

[34] Liò P. Wavelets in bioinformatics and computational biology: state of art and perspectives. Bioinformatics. 2003;19(1):2–9. doi: 10.1093/bioinformatics/19.1.2 12499286

[35] Meng T, Soliman AT, Shyu M-L, Yang Y, Chen S-C, Iyengar SS, et al. Wavelet analysis in current cancer genome research: a survey. IEEE/ACM Trans Comput Biol Bioinform. 2013;10(6):1442–59. doi: 10.1109/TCBB.2013.134 24407303

[36] Levo M, Raimundo J, Bing XY, Sisco Z, Batut PJ, Ryabichko S, et al. Transcriptional coupling of distant regulatory genes in living embryos. Nature. 2022;605(7911):754–60. doi: 10.1038/s41586-022-04680-7 35508662 PMC9886134

[37] Hug CB, Grimaldi AG, Kruse K, Vaquerizas JM. Chromatin architecture emerges during zygotic genome activation independent of transcription. Cell. 2017;169(2):216-228.e19. doi: 10.1016/j.cell.2017.03.024 28388407

[38] Lee D-S, Luo C, Zhou J, Chandran S, Rivkin A, Bartlett A, et al. Simultaneous profiling of 3D genome structure and DNA methylation in single human cells. Nat Methods. 2019;16(10):999–1006. doi: 10.1038/s41592-019-0547-z 31501549 PMC6765423

[39] Krietenstein N, Abraham S, Venev SV, Abdennur N, Gibcus J, Hsieh T-HS, et al. Ultrastructural details of mammalian chromosome architecture. Mol Cell. 2020;78(3):554-565.e7. doi: 10.1016/j.molcel.2020.03.003 32213324 PMC7222625

[40] Sefer E. A comparison of topologically associating domain callers over mammals at high resolution. BMC Bioinform. 2022;23(1):127. doi: 10.1186/s12859-022-04674-2 35413815 PMC9006547

[41] Nagano T, Lubling Y, Várnai C, Dudley C, Leung W, Baran Y, et al. Cell-cycle dynamics of chromosomal organization at single-cell resolution. Nature. 2017;547(7661):61–7. doi: 10.1038/nature23001 28682332 PMC5567812

[42] Olivares-Chauvet P, Mukamel Z, Lifshitz A, Schwartzman O, Elkayam NO, Lubling Y, et al. Capturing pairwise and multi-way chromosomal conformations using chromosomal walks. Nature. 2016;540(7632):296–300. doi: 10.1038/nature20158 27919068

[43] Li L, Lyu X, Hou C, Takenaka N, Nguyen HQ, Ong C-T, et al. Widespread rearrangement of 3D chromatin organization underlies polycomb-mediated stress-induced silencing. Mol Cell. 2015;58(2):216–31. doi: 10.1016/j.molcel.2015.02.023 25818644 PMC4402144

[44] Ray J, Munn PR, Vihervaara A, Lewis JJ, Ozer A, Danko CG, et al. Chromatin conformation remains stable upon extensive transcriptional changes driven by heat shock. Proc Natl Acad Sci U S A. 2019;116(39):19431–9. doi: 10.1073/pnas.1901244116 31506350 PMC6765289

[45] Zhang Y, Li T, Preissl S, Amaral ML, Grinstein JD, Farah EN, et al. Transcriptionally active HERV-H retrotransposons demarcate topologically associating domains in human pluripotent stem cells. Nat Genet. 2019;51(9):1380–8. doi: 10.1038/s41588-019-0479-7 31427791 PMC6722002

[46] Wang S, Su J-H, Beliveau BJ, Bintu B, Moffitt JR, Wu C, et al. Spatial organization of chromatin domains and compartments in single chromosomes. Science. 2016;353(6299):598–602. doi: 10.1126/science.aaf8084 27445307 PMC4991974

[47] Du Z, Zheng H, Huang B, Ma R, Wu J, Zhang X, et al. Allelic reprogramming of 3D chromatin architecture during early mammalian development. Nature. 2017;547(7662):232–5. doi: 10.1038/nature23263 28703188

[48] Ke Y, Xu Y, Chen X, Feng S, Liu Z, Sun Y, et al. 3D chromatin structures of mature gametes and structural reprogramming during mammalian embryogenesis. Cell. 2017;170(2):367-381.e20. doi: 10.1016/j.cell.2017.06.029 28709003

[49] Zheng H, Xie W. The role of 3D genome organization in development and cell differentiation. Nat Rev Mol Cell Biol. 2019;20(9):535–50. doi: 10.1038/s41580-019-0132-4 31197269

[50] Vallot A, Tachibana K. The emergence of genome architecture and zygotic genome activation. Curr Opin Cell Biol. 2020;64:50–7. doi: 10.1016/j.ceb.2020.02.002 32220807 PMC7374442

[51] Jukam D, Shariati SAM, Skotheim JM. Zygotic genome activation in vertebrates. Dev Cell. 2017;42(4):316–32. doi: 10.1016/j.devcel.2017.07.026 28829942 PMC5714289

[52] Cherbas L, Willingham A, Zhang D, Yang L, Zou Y, Eads BD, et al. The transcriptional diversity of 25 Drosophila cell lines. Genome Res. 2011;21(2):301–14. doi: 10.1101/gr.112961.110 21177962 PMC3032933

[53] Gabriele M, Brandão HB, Grosse-Holz S, Jha A, Dailey GM, Cattoglio C, et al. Dynamics of CTCF- and cohesin-mediated chromatin looping revealed by live-cell imaging. Science. 2022;376(6592):496–501. doi: 10.1126/science.abn6583 35420890 PMC9069445

[54] Nanni L, Ceri S, Logie C. Spatial patterns of CTCF sites define the anatomy of TADs and their boundaries. Genome Biol. 2020;21(1):197. doi: 10.1186/s13059-020-02108-x 32782014 PMC7422557

[55] Dixon JR, Gorkin DU, Ren B. Chromatin domains: the unit of chromosome organization. Mol Cell. 2016;62(5):668–80. doi: 10.1016/j.molcel.2016.05.018 27259200 PMC5371509

[56] Rao SSP, Huntley MH, Durand NC, Stamenova EK, Bochkov ID, Robinson JT, et al. A 3D map of the human genome at kilobase resolution reveals principles of chromatin looping. Cell. 2014;159(7):1665–80. doi: 10.1016/j.cell.2014.11.021 25497547 PMC5635824

[57] Sanborn AL, Rao SSP, Huang S-C, Durand NC, Huntley MH, Jewett AI, et al. Chromatin extrusion explains key features of loop and domain formation in wild-type and engineered genomes. Proc Natl Acad Sci U S A. 2015;112(47):E6456-65. doi: 10.1073/pnas.1518552112 26499245 PMC4664323

[58] Alipour E, Marko JF. Self-organization of domain structures by DNA-loop-extruding enzymes. Nucleic Acids Res. 2012;40(22):11202–12. doi: 10.1093/nar/gks925 23074191 PMC3526278

[59] Dorsett D. The many roles of cohesin in drosophila gene transcription. Trends Genet. 2019;35(7):542–51. doi: 10.1016/j.tig.2019.04.002 31130395 PMC6571051

[60] Fudenberg G, Imakaev M, Lu C, Goloborodko A, Abdennur N, Mirny LA. Formation of chromosomal domains by loop extrusion. Cell Rep. 2016;15(9):2038–49. doi: 10.1016/j.celrep.2016.04.085 27210764 PMC4889513

[61] Wendt KS, Yoshida K, Itoh T, Bando M, Koch B, Schirghuber E, et al. Cohesin mediates transcriptional insulation by CCCTC-binding factor. Nature. 2008;451(7180):796–801. doi: 10.1038/nature06634 18235444

[62] de Wit E, Vos ESM, Holwerda SJB, Valdes-Quezada C, Verstegen MJAM, Teunissen H, et al. CTCF binding polarity determines chromatin looping. Mol Cell. 2015;60(4):676–84. doi: 10.1016/j.molcel.2015.09.023 26527277

[63] Nichols MH, Corces VG. A CTCF code for 3D genome architecture. Cell. 2015;162(4):703–5. doi: 10.1016/j.cell.2015.07.053 26276625 PMC4745123

[64] Ong C-T, Corces VG. CTCF: an architectural protein bridging genome topology and function. Nat Rev Genet. 2014;15(4):234–46. doi: 10.1038/nrg3663 24614316 PMC4610363

[65] Phillips JE, Corces VG. CTCF: master weaver of the genome. Cell. 2009;137(7):1194–211. doi: 10.1016/j.cell.2009.06.001 19563753 PMC3040116

[66] Dekker J, Belmont AS, Guttman M, Leshyk VO, Lis JT, Lomvardas S, et al. The 4D nucleome project. Nature. 2017;549(7671):219–26. doi: 10.1038/nature23884 28905911 PMC5617335

[67] Zhang J, Lee D, Dhiman V, Jiang P, Xu J, McGillivray P, et al. An integrative ENCODE resource for cancer genomics. Nat Commun. 2020;11(1):3696. doi: 10.1038/s41467-020-14743-w 32728046 PMC7391744

[68] Liu M, Maurano MT, Wang H, Qi H, Song C-Z, Navas PA, et al. Genomic discovery of potent chromatin insulators for human gene therapy. Nat Biotechnol. 2015;33(2):198–203. doi: 10.1038/nbt.3062 25580597

[69] ENCODE Project Consortium. An integrated encyclopedia of DNA elements in the human genome. Nature. 2012;489(7414):57–74. doi: 10.1038/nature11247 22955616 PMC3439153

[70] Akgol Oksuz B, Yang L, Abraham S, Venev SV, Krietenstein N, Parsi KM, et al. Systematic evaluation of chromosome conformation capture assays. Nat Methods. 2021;18(9):1046–55. doi: 10.1038/s41592-021-01248-7 34480151 PMC8446342

[71] Rowley MJ, Nichols MH, Lyu X, Ando-Kuri M, Rivera ISM, Hermetz K, et al. Evolutionarily conserved principles predict 3D chromatin organization. Mol Cell. 2017;67(5):837-852.e7. doi: 10.1016/j.molcel.2017.07.022 28826674 PMC5591081

[72] Van Bortle K, Ramos E, Takenaka N, Yang J, Wahi JE, Corces VG. Drosophila CTCF tandemly aligns with other insulator proteins at the borders of H3K27me3 domains. Genome Res. 2012;22(11):2176–87. doi: 10.1101/gr.136788.111 22722341 PMC3483547

[73] Cubenas-Potts C, Rowley MJ, Lyu X, Li G, Lei EP, Corces VG. Different enhancer classes in Drosophila bind distinct architectural proteins and mediate unique chromatin interactions and 3D architecture. Nucleic Acids Res. 2017;45(4):1714–30.doi: 10.1093/nar/gkw1114 27899590 PMC5389536

[74] Sexton T, Yaffe E, Kenigsberg E, Bantignies F, Leblanc B, Hoichman M, et al. Three-dimensional folding and functional organization principles of the Drosophila genome. Cell. 2012;148(3):458–72. doi: 10.1016/j.cell.2012.01.010 22265598

[75] Hornuss D, Lange B, Schröter N, Rieg S, Kern WV, Wagner D. Anosmia in COVID-19 patients. Clin Microbiol Infect. 2020;26(10):1426–7. doi: 10.1016/j.cmi.2020.05.017 32447049 PMC7242197

[76] Shamsundara M, Jayalakshmi L. Anosmia-an effect of COVID-19 infection-review. Indian J Otolaryngol Head Neck Surg. 2023;75(Suppl 1):815–21. doi: 10.1007/s12070-022-03401-w 36593947 PMC9798353

[77] Callender LA, Curran M, Bates SM, Mairesse M, Weigandt J, Betts CJ. The impact of pre-existing comorbidities and therapeutic interventions on COVID-19. Front Immunol. 2020;11:1991. doi: 10.3389/fimmu.2020.01991 32903476 PMC7437504

[78] Meng X, Deng Y, Dai Z, Meng Z. COVID-19 and anosmia: a review based on up-to-date knowledge. Am J Otolaryngol. 2020;41(5):102581. doi: 10.1016/j.amjoto.2020.102581 32563019 PMC7265845

[79] Zazhytska M, Kodra A, Hoagland DA, Frere J, Fullard JF, Shayya H, et al. Non-cell-autonomous disruption of nuclear architecture as a potential cause of COVID-19-induced anosmia. Cell. 2022;185(6):1052-1064.e12. doi: 10.1016/j.cell.2022.01.024 35180380 PMC8808699

[80] Singh A, Agrawal AF. Two forms of sexual dimorphism in gene expression in drosophila melanogaster: their coincidence and evolutionary genetics. Mol Biol Evol. 2023;40(5):msad091. doi: 10.1093/molbev/msad091 37116199 PMC10162685

[81] Ellegren H, Parsch J. The evolution of sex-biased genes and sex-biased gene expression. Nat Rev Genet. 2007;8(9):689–98. doi: 10.1038/nrg2167 17680007

[82] Asahina K. Sex differences in drosophila behavior: qualitative and quantitative dimorphism. Curr Opin Physiol. 2018;6:35–45. doi: 10.1016/j.cophys.2018.04.004 30386833 PMC6205217

[83] Yamamoto D, Koganezawa M. Genes and circuits of courtship behaviour in Drosophila males. Nat Rev Neurosci. 2013;14(10):681–92. doi: 10.1038/nrn3567 24052176

[84] Llopart A, Brud E, Pettie N, Comeron JM. Support for the dominance theory in drosophila transcriptomes. Genetics. 2018;210(2):703–18. doi: 10.1534/genetics.118.301229 30131345 PMC6216581

[85] Ghavi-Helm Y, Jankowski A, Meiers S, Viales RR, Korbel JO, Furlong EEM. Highly rearranged chromosomes reveal uncoupling between genome topology and gene expression. Nat Genet. 2019;51(8):1272–82. doi: 10.1038/s41588-019-0462-3 31308546 PMC7116017

[86] Li H, Durbin R. Fast and accurate short read alignment with Burrows-Wheeler transform. Bioinformatics. 2009;25(14):1754–60. doi: 10.1093/bioinformatics/btp324 19451168 PMC2705234

[87] Ramírez F, Bhardwaj V, Arrigoni L, Lam KC, Grüning BA, Villaveces J, et al. High-resolution TADs reveal DNA sequences underlying genome organization in flies. Nat Commun. 2018;9(1):189. doi: 10.1038/s41467-017-02525-w 29335486 PMC5768762

[88] Abdennur N, Mirny LA. Cooler: scalable storage for Hi-C data and other genomically labeled arrays. Bioinformatics. 2020;36(1):311–6. doi: 10.1093/bioinformatics/btz540 31290943 PMC8205516

[89] Quinlan AR, Hall IM. BEDTools: a flexible suite of utilities for comparing genomic features. Bioinformatics. 2010;26(6):841–2. doi: 10.1093/bioinformatics/btq033 20110278 PMC2832824

[90] Aldrich E. Wavelets: a package of functions for computing wavelet filters, wavelet transforms and multiresolution analyses. 2013.

[91] Percival DP. On estimation of the wavelet variance. Biometrika. 1995;82(3):619–31. doi: 10.1093/biomet/82.3.619

[92] Serroukh A, Walden AT, Percival DB. Statistical properties and uses of the wavelet variance estimator for the scale analysis of time series. J Am Stat Assoc. 2000;95(449):184–96. doi: 10.1080/01621459.2000.10473913

[93] Donoho DL, Johnstone IM. Ideal spatial adaptation by wavelet shrinkage. Biometrika. 1994;81(3):425–55. doi: 10.1093/biomet/81.3.425

[94] Wolff J, Rabbani L, Gilsbach R, Richard G, Manke T, Backofen R, et al. Galaxy HiCExplorer 3: a web server for reproducible Hi-C, capture Hi-C and single-cell Hi-C data analysis, quality control and visualization. Nucleic Acids Res. 2020;48(W1):W177–84. doi: 10.1093/nar/gkaa220 32301980 PMC7319437

[95] Durand NC, Shamim MS, Machol I, Rao SSP, Huntley MH, Lander ES, et al. Juicer provides a one-click system for analyzing loop-resolution Hi-C experiments. Cell Syst. 2016;3(1):95–8. doi: 10.1016/j.cels.2016.07.002 27467249 PMC5846465

[96] An L, Yang T, Yang J, Nuebler J, Xiang G, Hardison RC, et al. OnTAD: hierarchical domain structure reveals the divergence of activity among TADs and boundaries. Genome Biol. 2019;20(1):282. doi: 10.1186/s13059-019-1893-y 31847870 PMC6918570

[97] Shin H, Shi Y, Dai C, Tjong H, Gong K, Alber F, et al. TopDom: an efficient and deterministic method for identifying topological domains in genomes. Nucleic Acids Res. 2016;44(7):e70. doi: 10.1093/nar/gkv1505 26704975 PMC4838359

[98] Lyu H, Li L, Wu Z, Wang T, Zheng J, Wang H. TADBD: a sensitive and fast method for detection of typologically associated domain boundaries. Biotechniques. 2020;69(1):376–83. doi: 10.2144/btn-2019-0165 32252545

[99] Chen F, Li G, Zhang MQ, Chen Y. HiCDB: a sensitive and robust method for detecting contact domain boundaries. Nucleic Acids Res. 2018;46(21):11239–50. doi: 10.1093/nar/gky789 30184171 PMC6265446

[100] Roayaei Ardakany A, Lonardi S. Efficient and accurate detection of topologically associating domains from contact maps. In: 17th international workshop on algorithms in bioinformatics (WABI 2017). 2017.

[101] Oluwadare O, Cheng J. ClusterTAD: an unsupervised machine learning approach to detecting topologically associated domains of chromosomes from Hi-C data. BMC Bioinformatics. 2017;18(1):480. doi: 10.1186/s12859-017-1931-2 29137603 PMC5686814

[102] Norton HK, Emerson DJ, Huang H, Kim J, Titus KR, Gu S, et al. Detecting hierarchical genome folding with network modularity. Nat Methods. 2018;15(2):119–22. doi: 10.1038/nmeth.4560 29334377 PMC6029251

[103] Filippova D, Patro R, Duggal G, Kingsford C. Identification of alternative topological domains in chromatin. Algorithms Mol Biol. 2014;9:14. doi: 10.1186/1748-7188-9-14 24868242 PMC4019371

[104] Crane E, Bian Q, McCord RP, Lajoie BR, Wheeler BS, Ralston EJ, et al. Condensin-driven remodelling of X chromosome topology during dosage compensation. Nature. 2015;523(7559):240–4. doi: 10.1038/nature14450 26030525 PMC4498965

[105] Cresswell KG, Stansfield JC, Dozmorov MG. SpectralTAD: an R package for defining a hierarchy of topologically associated domains using spectral clustering. BMC Bioinformatics. 2020;21(1):319. doi: 10.1186/s12859-020-03652-w 32689928 PMC7372752

[106] Zhang YW, Wang MB, Li SC. SuperTAD: robust detection of hierarchical topologically associated domains with optimized structural information. Genome Biol. 2021;22(1):45. doi: 10.1186/s13059-020-02234-6 33494803 PMC7831269

[107] Lévy-Leduc C, Delattre M, Mary-Huard T, Robin S. Two-dimensional segmentation for analyzing Hi-C data. Bioinformatics. 2014;30(17):i386-92. doi: 10.1093/bioinformatics/btu443 25161224 PMC4147896

[108] Haddad N, Vaillant C, Jost D. IC-Finder: inferring robustly the hierarchical organization of chromatin folding. Nucleic Acids Res. 2017;45(10):e81. doi: 10.1093/nar/gkx036 28130423 PMC5449546

[109] Dixon JR, Selvaraj S, Yue F, Kim A, Li Y, Shen Y, et al. Topological domains in mammalian genomes identified by analysis of chromatin interactions. Nature. 2012;485(7398):376–80. doi: 10.1038/nature11082 22495300 PMC3356448

[110] Love MI, Huber W, Anders S. Moderated estimation of fold change and dispersion for RNA-seq data with DESeq2. Genome Biol. 2014;15(12):550. doi: 10.1186/s13059-014-0550-8 25516281 PMC4302049

[111] Tenenbaum D, RUnit S, Maintainer MBP, Carlson M, biocViews AP, ThirdPartyClient K. Package ‘keggrest’. Vienna, Austria: R Foundation for Statistical Computing; 2019.

