# Supplementary material for: A wavelet-based approach generates quantitative, scale-free and hierarchical descriptions of 3D genome structures and new biological insights
Source: PLoS Comput Biol. 2026 Jan 20;22(1):e1013887. doi: 10.1371/journal.pcbi.1013887 (PMC12829961; doi:10.1371/journal.pcbi.1013887)
Supplement: S3 Fig — (PDF) [file pcbi.1013887.s005.pdf]

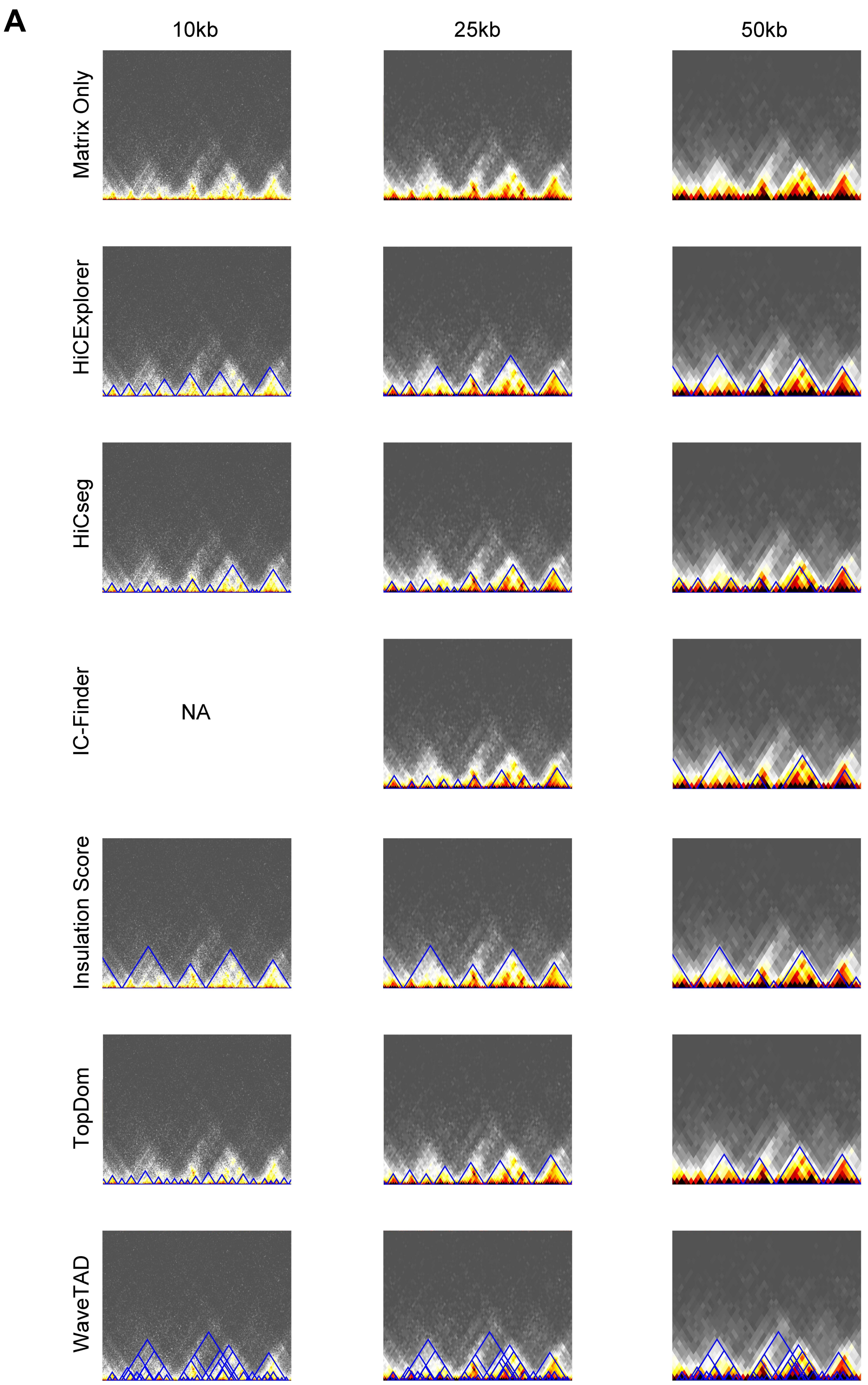

S3 Figure. TADs called by various TAD callers across resolutions for *Homo sapiens*. The "Matrix Only" heat maps depict the contact matrix at each resolution (10kb, 25kb, 50kb). Heat maps are overlayed with the various tool calls (excluding WaveTAD) at each resolution (10kb, 25kb, 50kb). WaveTAD's calls were overlayed over each resolution. Only TADs within the genomic region were plotted. Blue lines indicate TADs called. (**A**) Non-hierarchical TAD callers. (**B**) Hierarchical TAD callers. Data from Krietenstein et al. (2020) H1 hESC Hi-C (chr5:140,000,000-142,000,000).

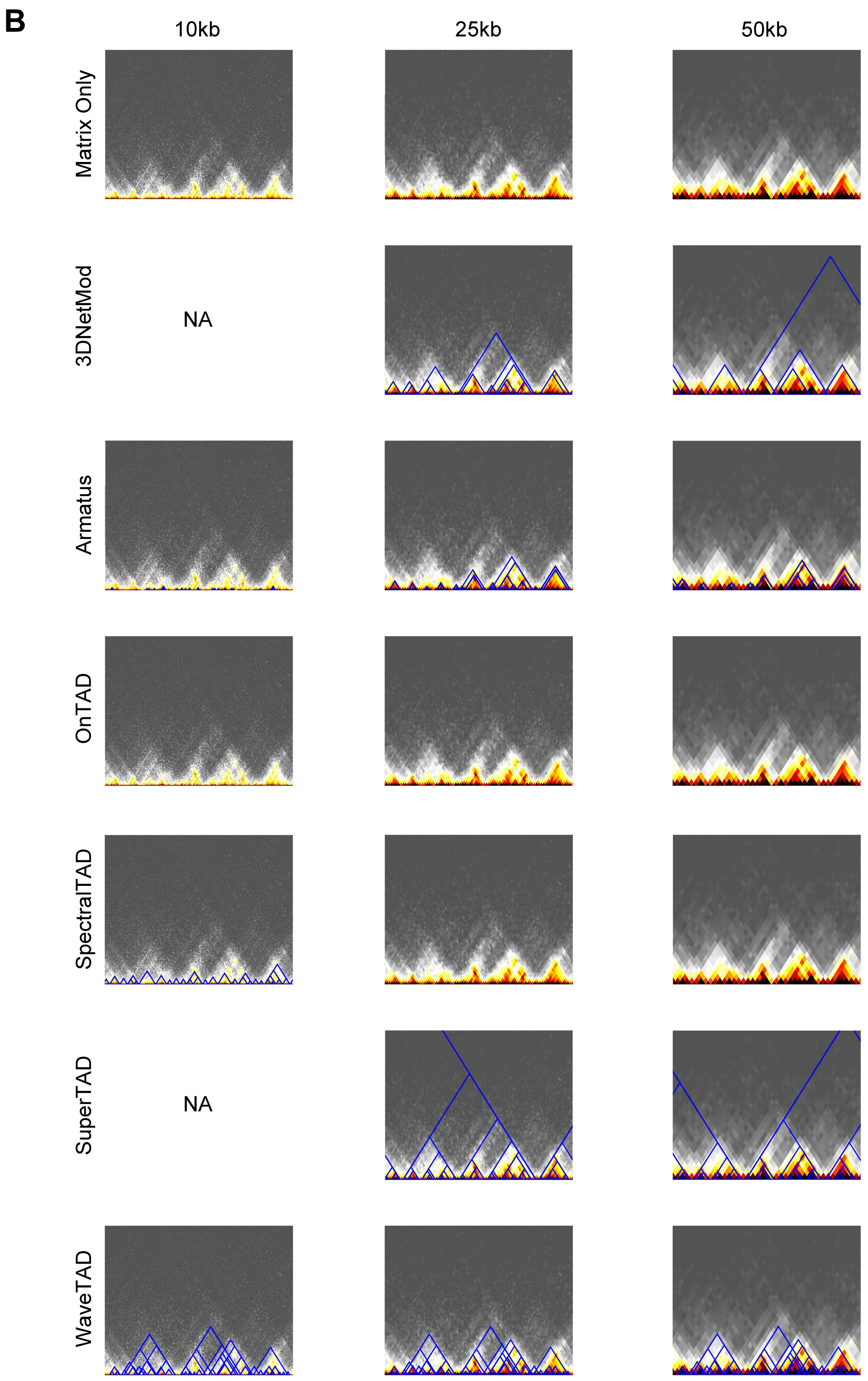

S3 Figure (cont).
