# Supplementary material for: A wavelet-based approach generates quantitative, scale-free and hierarchical descriptions of 3D genome structures and new biological insights
Source: PLoS Comput Biol. 2026 Jan 20;22(1):e1013887. doi: 10.1371/journal.pcbi.1013887 (PMC12829961; doi:10.1371/journal.pcbi.1013887)
Supplement: S4 Fig — (PDF) [file pcbi.1013887.s006.pdf]

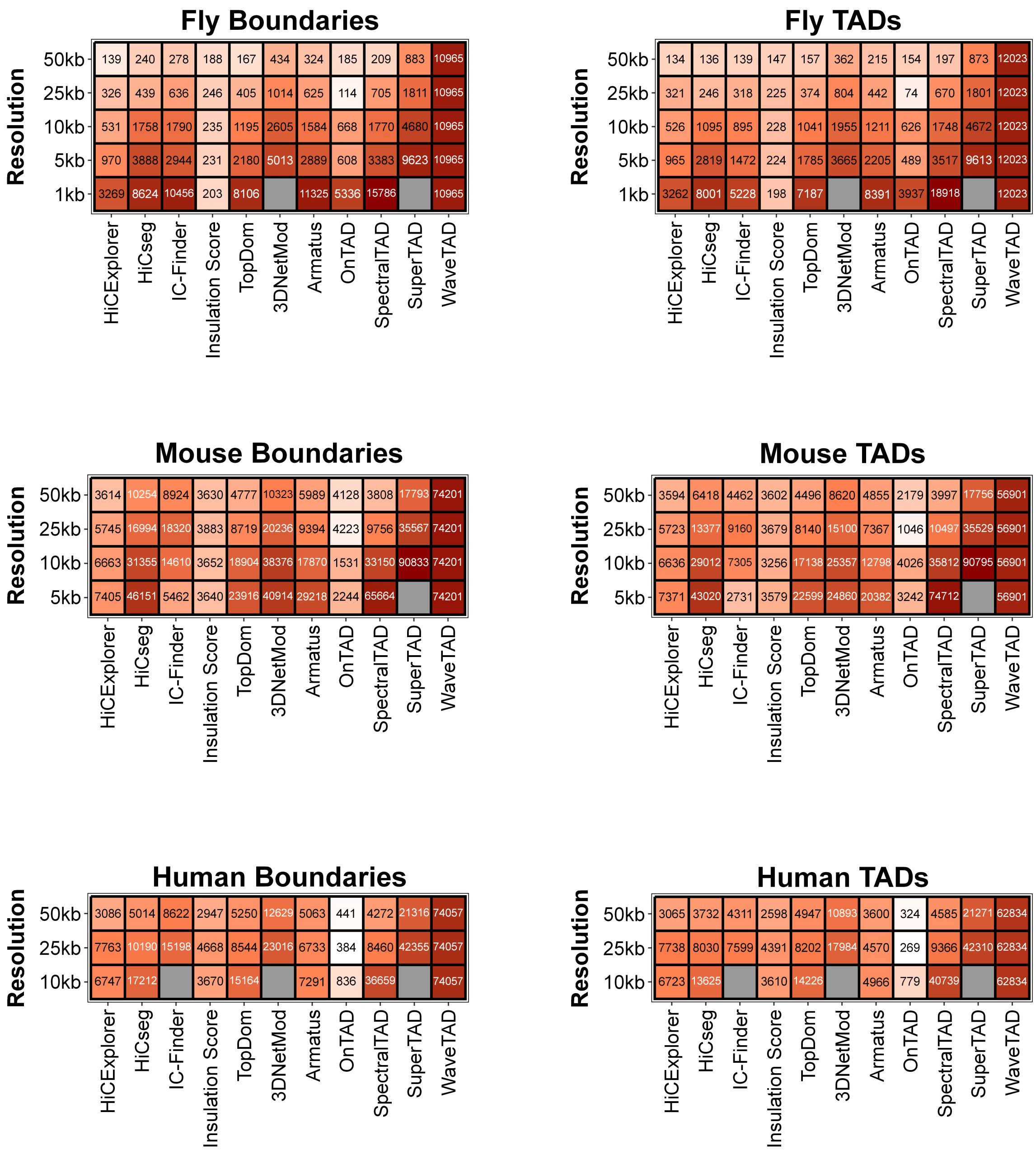

S4 Figure. Number of TAD and TAD boundary calls by various TAD callers across resolutions. Number of TAD and TAD boundaries called using different resolutions of contact matrices as the input for each species: fly (1kb, 5kb, 10kb, 25kb, 50kb), mouse (5kb, 10kb, 25kb, 50kb), and humans (10kb, 25kb, 50kb). WaveTAD calls are independent of resolution.
