# Supplementary material for: A wavelet-based approach generates quantitative, scale-free and hierarchical descriptions of 3D genome structures and new biological insights
Source: PLoS Comput Biol. 2026 Jan 20;22(1):e1013887. doi: 10.1371/journal.pcbi.1013887 (PMC12829961; doi:10.1371/journal.pcbi.1013887)
Supplement: S5 Fig — (PDF) [file pcbi.1013887.s007.pdf]

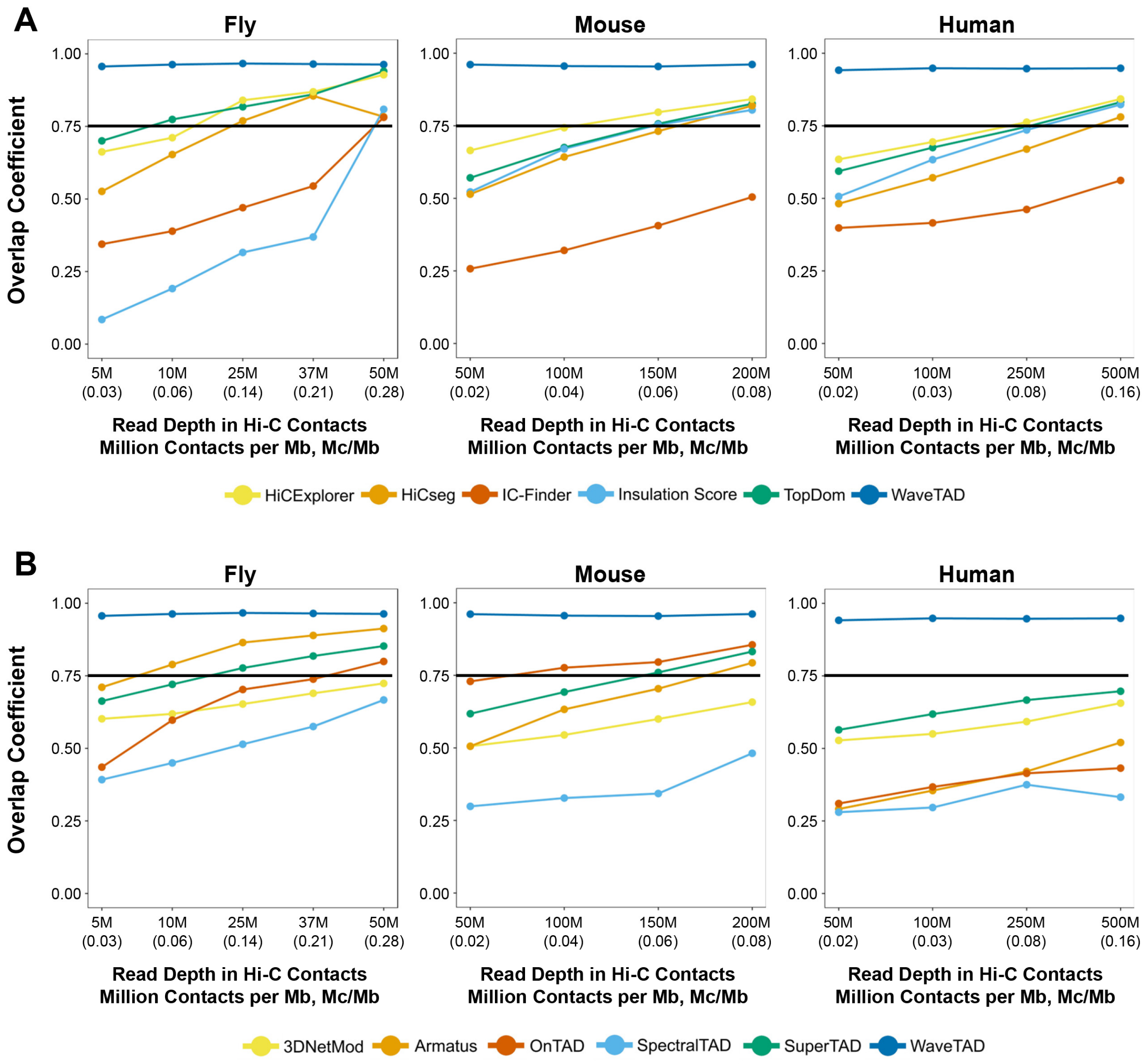

S5 Figure. Comparison of TAD calls between methods. Concordance of TAD boundaries using the overlap coefficient across read depths (number of million mapped Hi-C contacts, with million contacts per Mb is in parentheses) for non-hierarchical (**A**) and hierarchical (**B**) TAD callers. The overlap coefficient was estimated relative to the highest contact read depth for each species: 75M (0.35 Mc/Mb) for flies, 250M (0.10 Mc/Mb) for mouse, and 1B (0.31 Mc/Mb) for human. A resolution of 10kb for fly and 25kb for mouse and human was used.
