# Supplementary material for: A wavelet-based approach generates quantitative, scale-free and hierarchical descriptions of 3D genome structures and new biological insights
Source: PLoS Comput Biol. 2026 Jan 20;22(1):e1013887. doi: 10.1371/journal.pcbi.1013887 (PMC12829961; doi:10.1371/journal.pcbi.1013887)
Supplement: S6 Fig — (PDF) [file pcbi.1013887.s008.pdf]

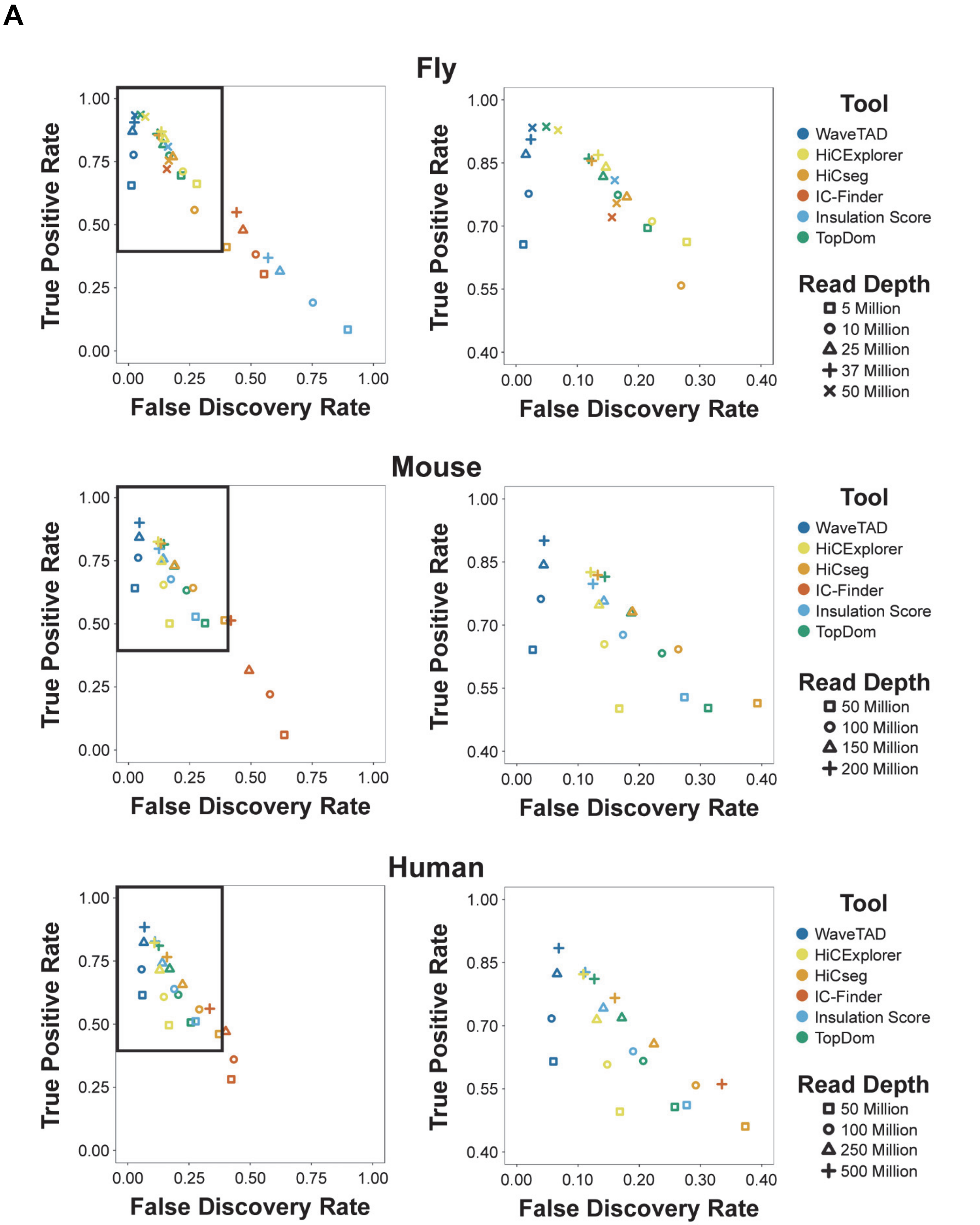

S6 Figure. True positive (TPR) and false discovery (FDR) rates of TAD boundaries called by various tools across read depths. The left panels display the complete spectrum of TPR and FDR, while the right panel zooms in on datapoints with a TPR greater than 0.4 and an FDR less than 0.4. The color represents the various tools, while the shape denotes read depth. TPR and FDR were determined relative to the highest contact read depth for each species: 75M (0.35 Mc/Mb) for flies, 250M (0.10 Mc/Mb) for mouse, and 1B (0.31 Mc/Mb) for human. (**A**) Non-hierarchical TAD callers. (**B**) Hierarchical TAD callers.

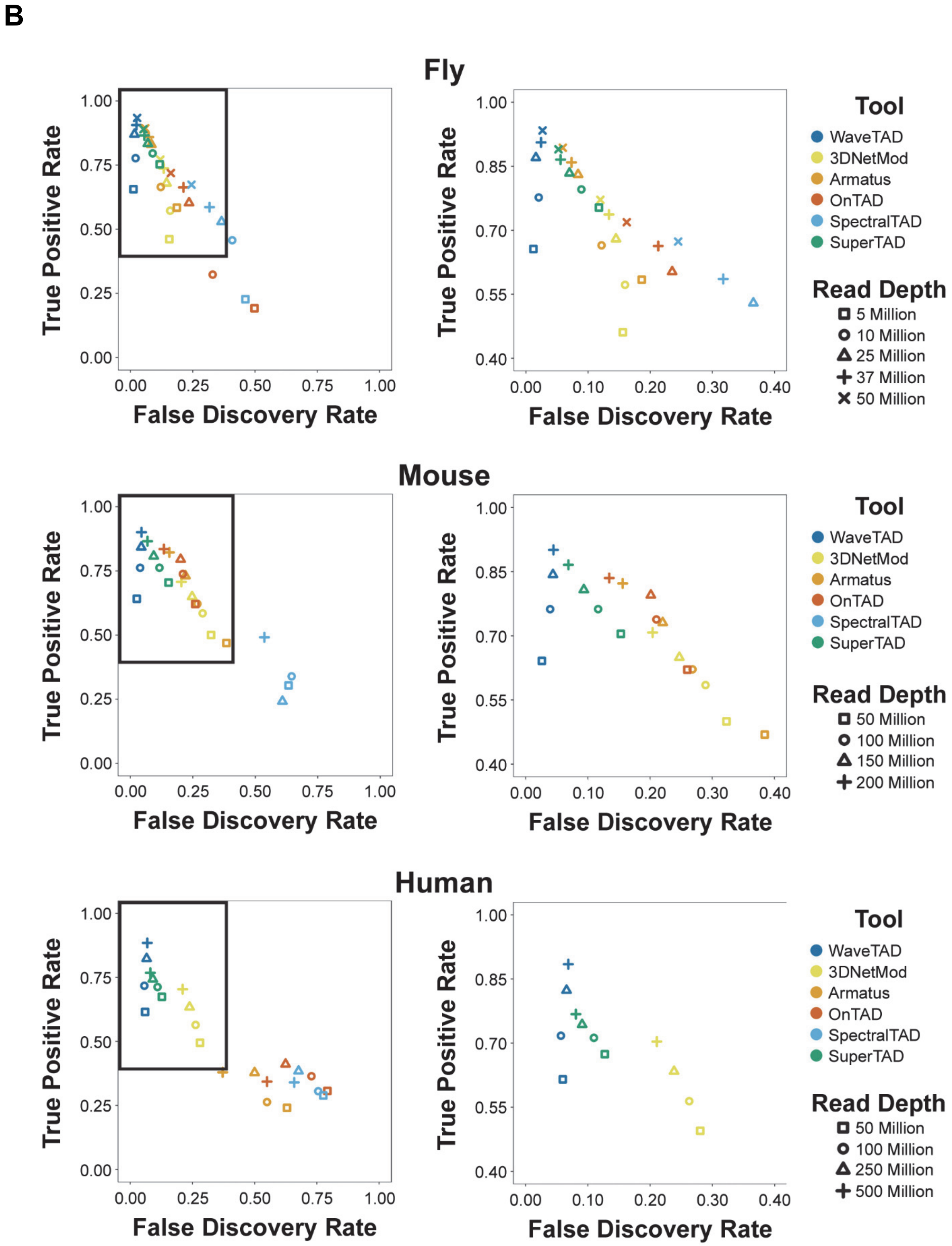

S6 Figure (cont).
