# Supplementary material for: A wavelet-based approach generates quantitative, scale-free and hierarchical descriptions of 3D genome structures and new biological insights
Source: PLoS Comput Biol. 2026 Jan 20;22(1):e1013887. doi: 10.1371/journal.pcbi.1013887 (PMC12829961; doi:10.1371/journal.pcbi.1013887)
Supplement: S7 Fig — (PDF) [file pcbi.1013887.s009.pdf]

A

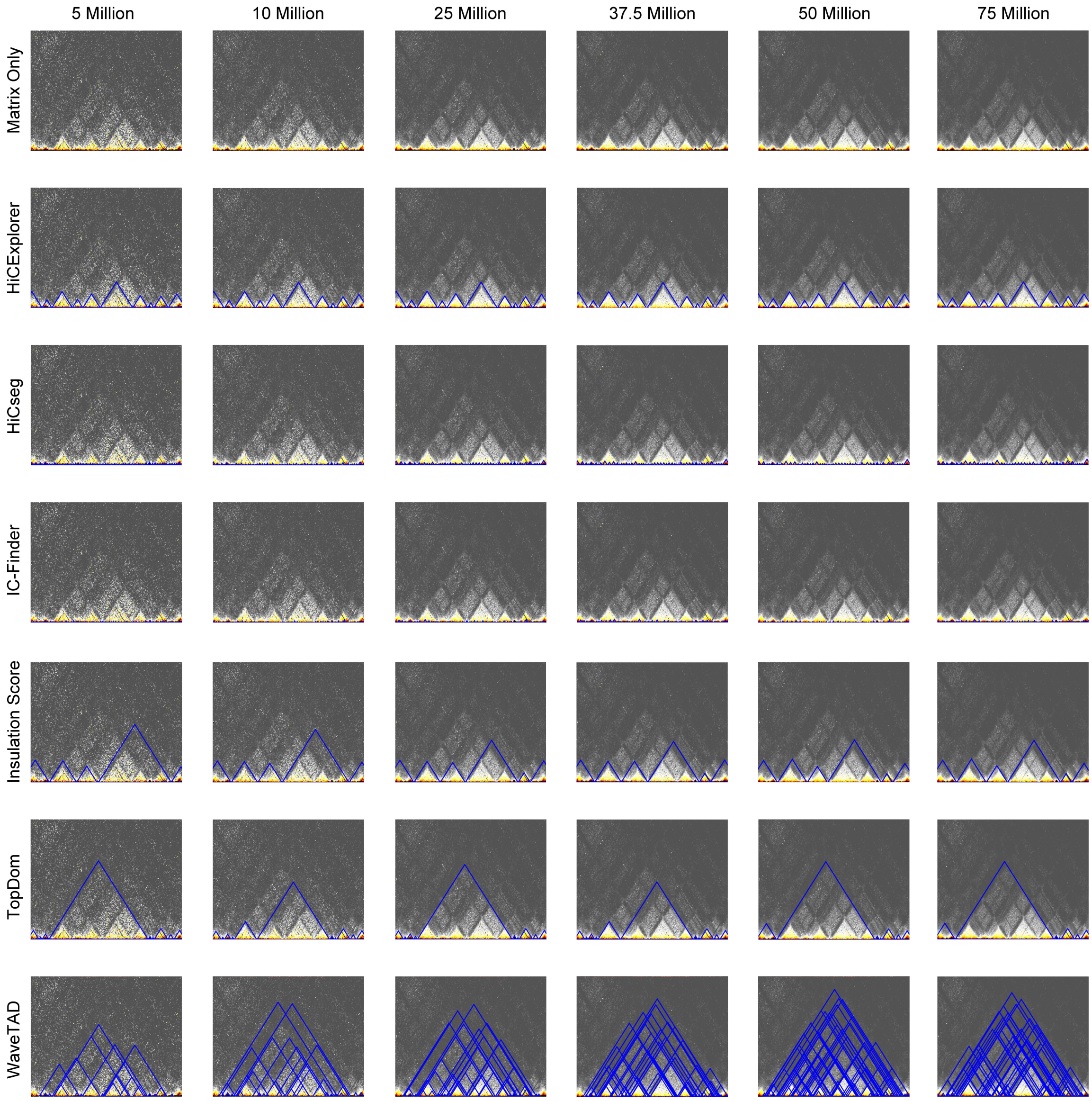

S7 Figure. TADs called by various TAD callers across read depths for *Drosophila melanogaster*. Heat maps of contacts matrices overlayed with the various tool calls at each read depth. Blue lines indicate TADs called. (**A**) Non-hierarchical TAD callers. (**B**) Hierarchical TAD callers. Data from Hug et al. (2017) staged embryos 3-4 hours post fertilization biological replicate 1 (chr3R:27,000,000-29,000,000) at 10kb resolution.

B

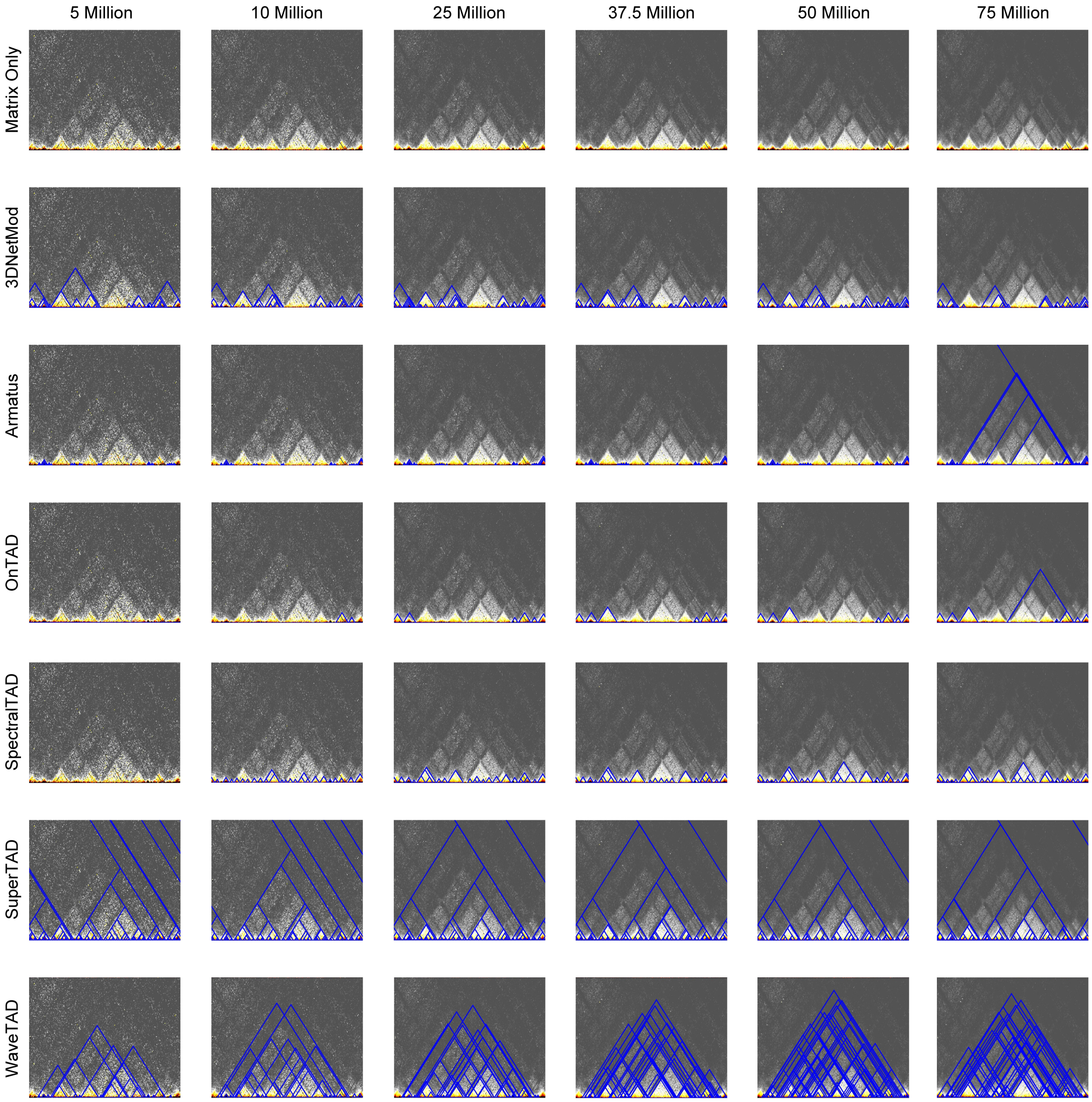

S7 Figure (cont).
