# Supplementary material for: A wavelet-based approach generates quantitative, scale-free and hierarchical descriptions of 3D genome structures and new biological insights
Source: PLoS Comput Biol. 2026 Jan 20;22(1):e1013887. doi: 10.1371/journal.pcbi.1013887 (PMC12829961; doi:10.1371/journal.pcbi.1013887)
Supplement: S8 Fig — (PDF) [file pcbi.1013887.s010.pdf]

A

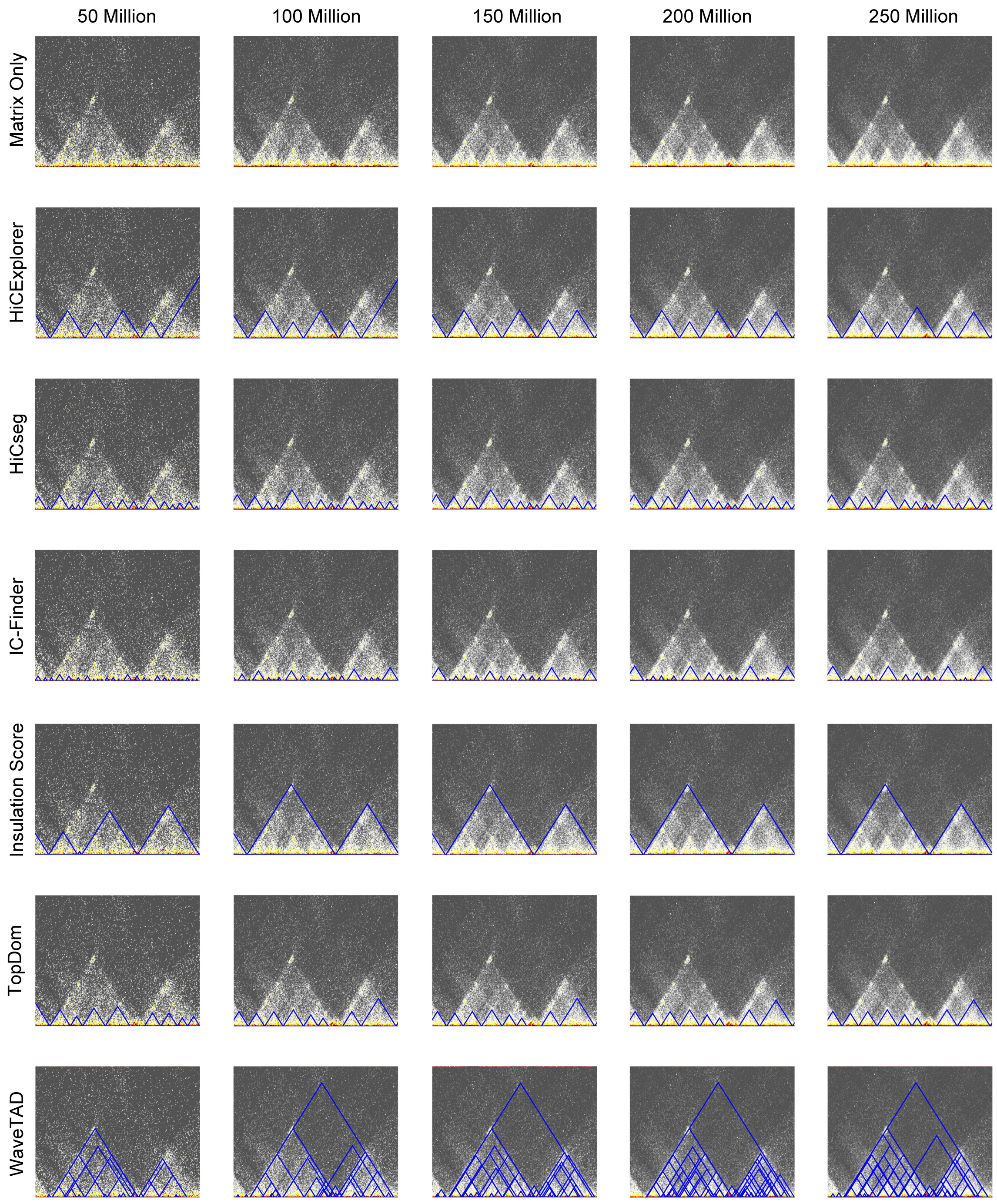

S8 Figure. TADs called by various TAD callers across read depths for *Mus musculus*. Heat maps of contacts matrices (10kb resolution) overlayed with the various tool calls at 25kb resolution for each read depth. Blue lines indicate TADs called. (**A**) Non-hierarchical TAD callers. (**B**) Hierarchical TAD callers. Data from Lee et al. (2019) mESC bulk Hi-C (chr6:51,000,000-53,000,000).

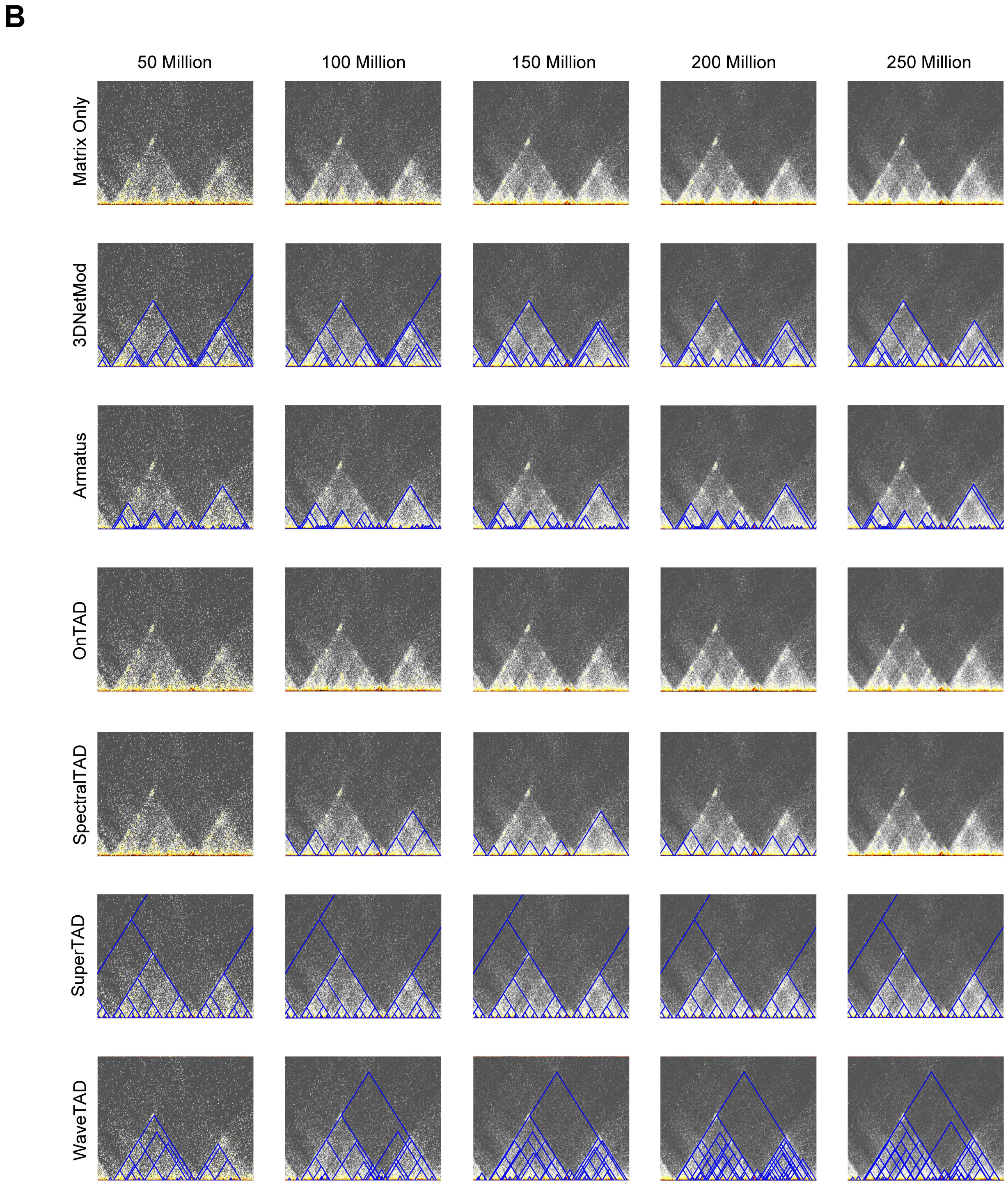

S8 Figure (cont).
