# Supplementary material for: A wavelet-based approach generates quantitative, scale-free and hierarchical descriptions of 3D genome structures and new biological insights
Source: PLoS Comput Biol. 2026 Jan 20;22(1):e1013887. doi: 10.1371/journal.pcbi.1013887 (PMC12829961; doi:10.1371/journal.pcbi.1013887)
Supplement: S10 Fig — (PDF) [file pcbi.1013887.s012.pdf]

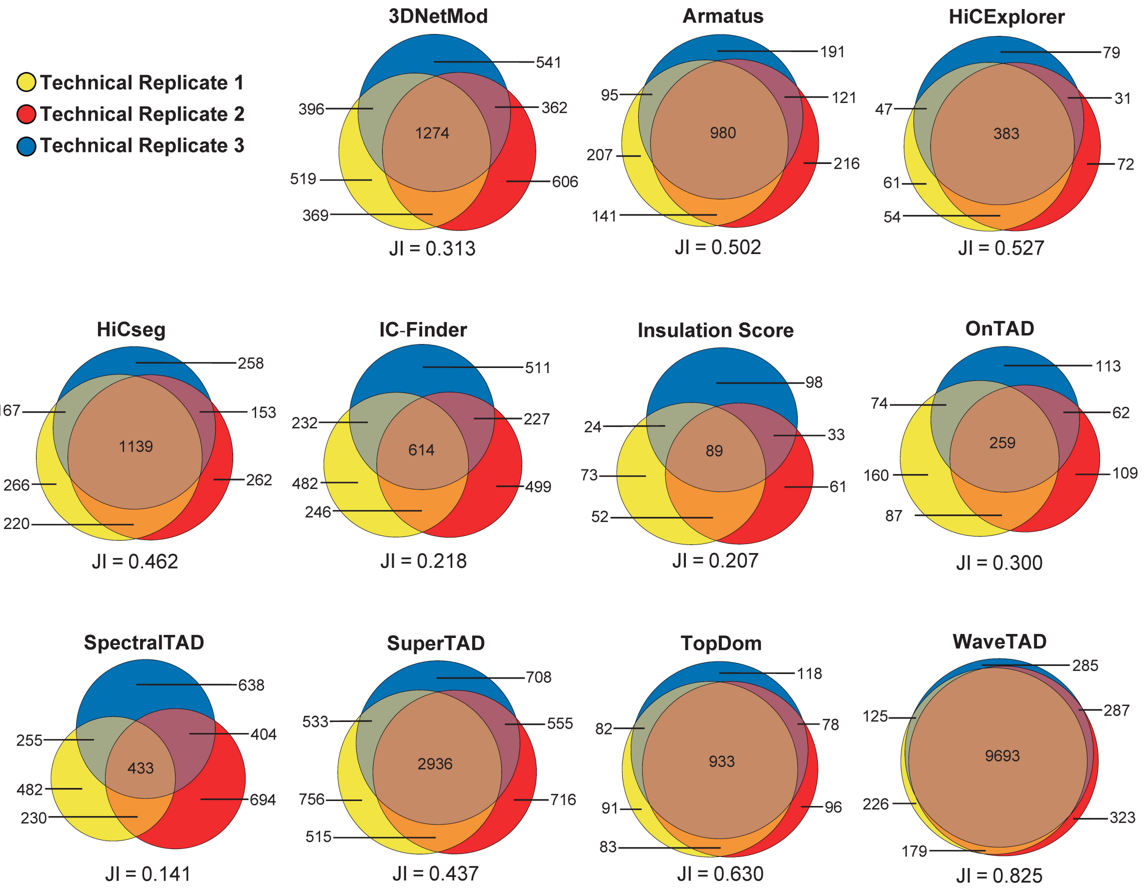

S10 Figure. Concordance of TADs called by various tools between technical replicates in *Drosophila melanogaster*. Venn diagram depicting the number of TADs and number of overlapping TADs called between technical replicates. For each tool the Jaccard Index for all the replicates is located below the Venn diagram. Data from Hug et al. (2017) staged embryos 3-4 hours post fertilization, biological replicate 1, technical replicates 1-3, (yellow, red, and blue, respectively).
