# Supplementary material for: A wavelet-based approach generates quantitative, scale-free and hierarchical descriptions of 3D genome structures and new biological insights
Source: PLoS Comput Biol. 2026 Jan 20;22(1):e1013887. doi: 10.1371/journal.pcbi.1013887 (PMC12829961; doi:10.1371/journal.pcbi.1013887)
Supplement: S11 Fig — (PDF) [file pcbi.1013887.s013.pdf]

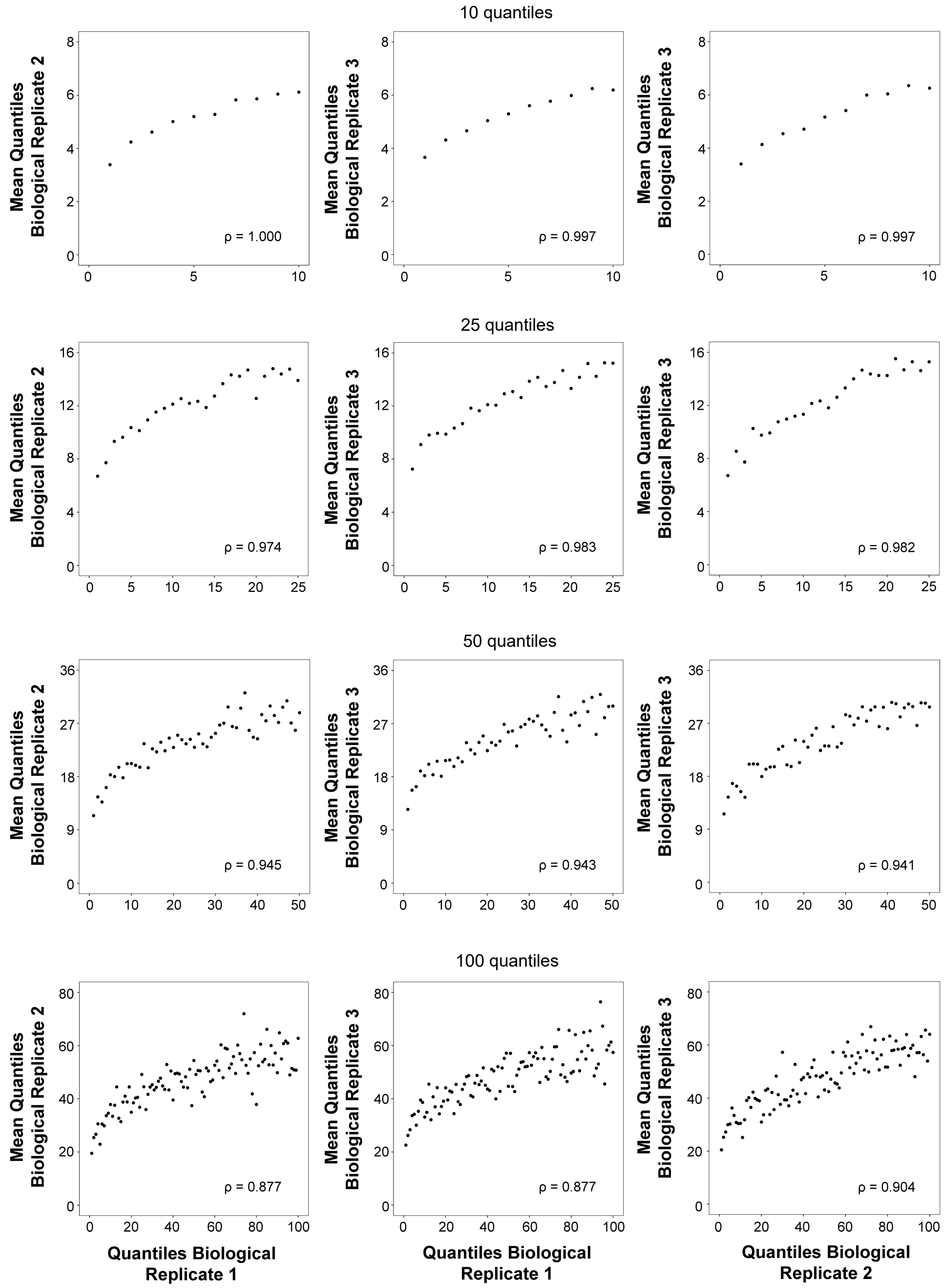

S11 Figure. WaveTAD probabilities are reproducible between biological replicates. Scatter plots depicting the relationship between the TAD probabilities across biological replicates. Each column of plots represents the comparisons between biological replicates (biological replicate 1 vs biological replicate 2, biological replicate 1 vs biological replicate 3, biological replicate 2 vs biological replicate 3). For each comparison, the *p*-values of all shared TADs were placed into either 10, 25, 50, or 100 quantiles (represented on the x-axis of each row of plots), where quantile 1 represents the quantile containing the weakest TADs and the largest quantile containing the strongest TADs. The y-axis of each plot represents the average quantile of *p*-values given the shared TADs in the given quantile of x-axis.
