# Supplementary material for: A wavelet-based approach generates quantitative, scale-free and hierarchical descriptions of 3D genome structures and new biological insights
Source: PLoS Comput Biol. 2026 Jan 20;22(1):e1013887. doi: 10.1371/journal.pcbi.1013887 (PMC12829961; doi:10.1371/journal.pcbi.1013887)
Supplement: S13 Fig — (PDF) [file pcbi.1013887.s015.pdf]

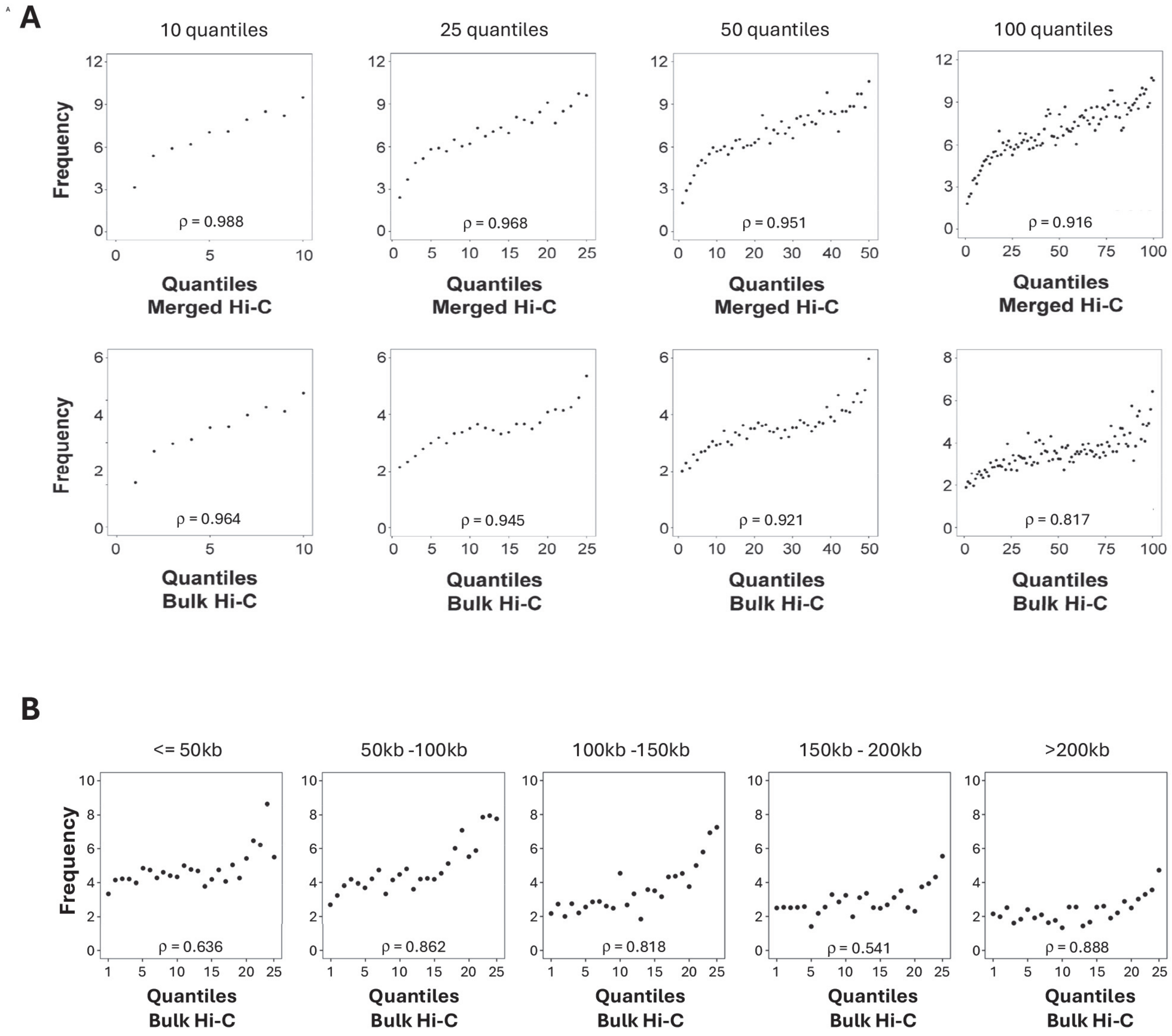

S13 Figure. TAD strengths generated by WaveTAD capture TAD frequency in heterogeneous samples. (**A**) Association (Spearman's Rank Correlation) between TAD strengths (x-axis) called in merged Hi-C or bulk Hi-C and the frequency of a TAD being called from single-nucleus Hi-C (snHi-C) (y-axis). For each comparison, both TAD strengths (probabilities) and the frequency of shared TADs were placed into either 10, 25, 50, or 100 quantiles to obtain averages. (**B**) Association when restricting the analysis to different TAD-size groups when applied to 25 quantiles from bulk Hi-C datasets.
