# Supplementary material for: A wavelet-based approach generates quantitative, scale-free and hierarchical descriptions of 3D genome structures and new biological insights
Source: PLoS Comput Biol. 2026 Jan 20;22(1):e1013887. doi: 10.1371/journal.pcbi.1013887 (PMC12829961; doi:10.1371/journal.pcbi.1013887)
Supplement: S14 Fig — (PDF) [file pcbi.1013887.s016.pdf]

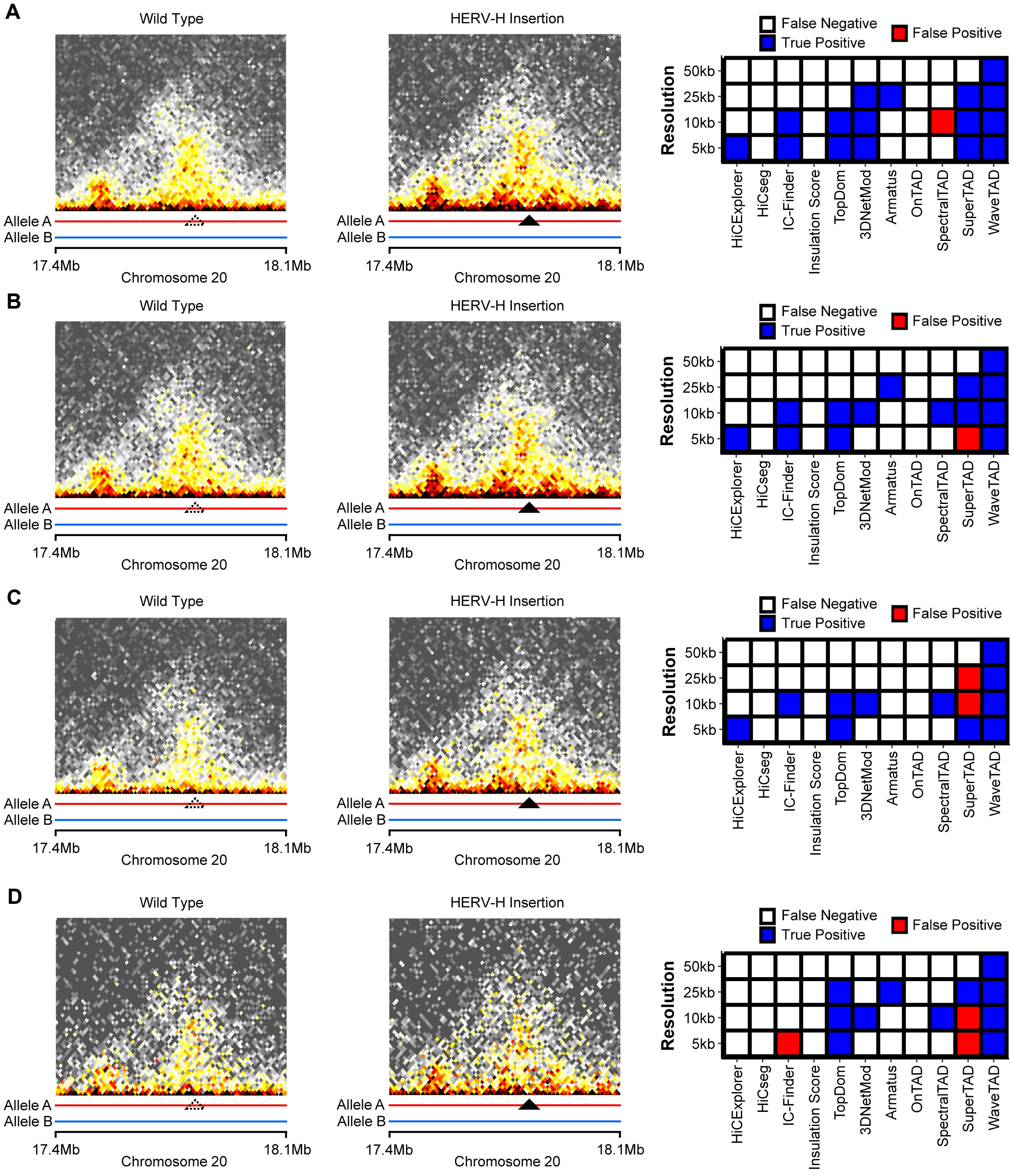

S14 Figure. WaveTAD calls TAD boundaries in cells with heterozygous alleles at low read depths. The first row of figures contains a contact matrix (10kb resolution) derived from a parental hESC cell line (wild type, left contact matrix), a contact matrix (10kb resolution) derived from an hESC cell line with a heterozygous TAD boundary cause by a de novo HERV-H insertion (right contact matrix), and a heat map illustrating the performance of TAD callers across resolutions and whether they correctly identify the heterozygous TAD boundary (blue), do not call the heterozygous TAD boundary (white), or incorrectly call a TAD boundary in the parental hESC cell line (red). Each row shows the two contact matrices and performance at different read depths. The read depth reflects the number of Hi-C contacts where both mates specifically map to chromosome 20. (**A**) full dataset, with 3.8 and 3.4 million contacts for wild type and HERV-H insertion, respectively. (**B**), (**C**) and (**D**) show results based on 3, 2 and 1 million contacts, respectively, in both samples. The region of interest shown is chr20:17,400,000-18,100,000.
