# Supplementary material for: A wavelet-based approach generates quantitative, scale-free and hierarchical descriptions of 3D genome structures and new biological insights
Source: PLoS Comput Biol. 2026 Jan 20;22(1):e1013887. doi: 10.1371/journal.pcbi.1013887 (PMC12829961; doi:10.1371/journal.pcbi.1013887)
Supplement: S15 Fig — (PDF) [file pcbi.1013887.s017.pdf]

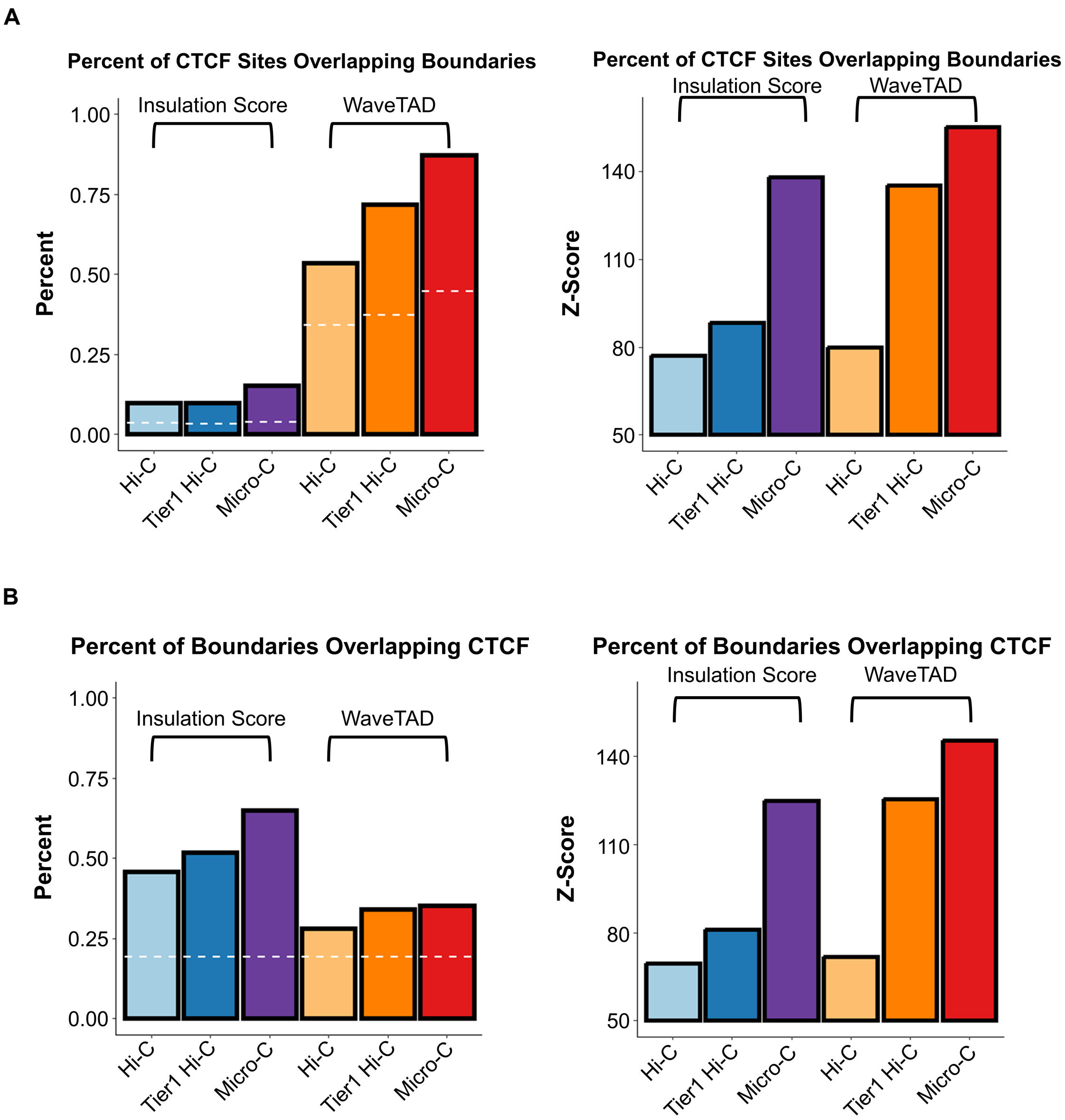

S15 Figure. CTCF sites and TAD stability. (**A**) Bar plots showing the percentage (left) and Z-scores (right) of CTCF sites overlapping TAD boundaries for both Insulation Score and WaveTAD for H1 hESC Hi-C, Tier1 Hi-C and Micro-C datasets. The dashed white line is the expected overlap when accounting for the different number of TAD boundaries called. (**B**) Bar plots showing the percent (left) and Z-scores (right) of TAD boundaries overlapping CTCF sites for both Insulation Score and WaveTAD for H1 hESC Hi-C, Tier1 Hi-C and Micro-C datasets. The dashed white line is the expectation a random boundary will overlap a CTCF site. The Z-scores were derived by bootstrapping.
