# Supplementary material for: A wavelet-based approach generates quantitative, scale-free and hierarchical descriptions of 3D genome structures and new biological insights
Source: PLoS Comput Biol. 2026 Jan 20;22(1):e1013887. doi: 10.1371/journal.pcbi.1013887 (PMC12829961; doi:10.1371/journal.pcbi.1013887)
Supplement: S16 Fig — (PDF) [file pcbi.1013887.s018.pdf]

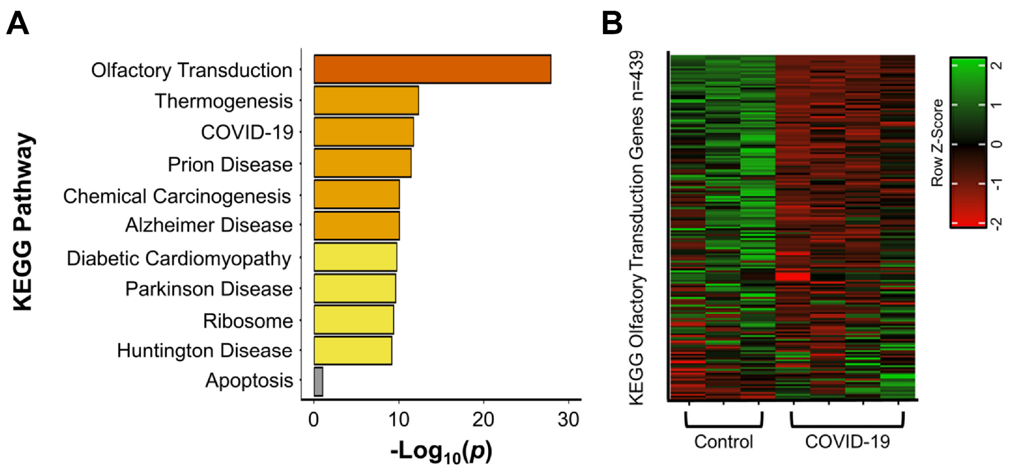

S16 Figure. SARS-CoV-2 infection downregulates olfactory transduction genes. (**A**) Bar plots depicting KEGG analysis for differentially expressed genes reveals enrichment for pathways associated with olfactory transduction (Mann-Whitney Test). (**B**) Heat map of differentially expressed olfactory transduction genes (N = 439) for three control and four COVID-19 samples. Colors represent the Z-scores (generated by DESeq2), with red being downregulated and green being upregulated.
